# Interbrain synchrony analysis for fNIRS: A signal processing primer

**DOI:** 10.1162/IMAG.a.1398

**Published:** 2026-10-29

**Authors:** Katarina Lodrant, Dimitri Van De Ville, Frank Scharnowski, Kathrin Kostorz

**Affiliations:** Department of Cognition, Emotion, and Methods in Psychology, Faculty of Psychology, University of Vienna, Vienna, Austria; Neuro-X Institute, Ecole Polytechnique Fédérale de Lausanne, Geneva, Switzerland; Department of Radiology and Medical Informatics, University of Geneva, Geneva, Switzerland

**Keywords:** interbrain synchrony, interpersonal neural synchrony, hyperscanning, fNIRS, time–frequency analysis, wavelet transform coherence

## Abstract

Interbrain synchrony (IBS), also referred to as interpersonal neural synchrony (INS), has become a central construct in social neuroscience and beyond. It describes the temporal alignment of neural activity across individuals and has been studied in a range of paradigms, including sequential interactions, shared-stimulus settings, and hyperscanning, that is, the simultaneous neuroimaging of two or more individuals during real-time interaction. Functional near-infrared spectroscopy (fNIRS) is increasingly used for such studies because it supports portable, naturalistic, face-to-face paradigms. A central goal in these experiments is to quantify IBS between interacting individuals. Wavelet transform coherence (WTC) has become the most widely used method for estimating IBS in fNIRS studies. However, its application involves several non-trivial signal-processing assumptions and time–frequency trade-offs that are often not explicitly addressed, making it difficult to apply and interpret the method consistently. Reliable estimation of IBS is crucial not only for experimental design but also for the interpretation and comparison of results within and across studies. Here, we aim to support this endeavor by providing a principled overview of the relevant signal-processing foundations. To enable researchers to make informed methodological choices, the paper first introduces key concepts in time–frequency analysis and clarifies their relation to WTC. These principles are then connected to the analysis of IBS in fNIRS datasets, with particular emphasis on the time–frequency constraints inherent to wavelet-based methods. Building on these foundations, we provide practical recommendations for experiment design, preprocessing, and frequency targeting. By making resolution limits and analysis assumptions explicit, this primer aims to support improved study planning and more consistent interpretation of IBS in the rapidly growing field of fNIRS-based investigations and beyond. Establishing a shared methodological foundation for IBS analysis is essential for advancing toward more rigorous, transparent, and reproducible measurement of interpersonal brain dynamics in naturalistic neuroscience.

## 1 Introduction

In recent years, social and cognitive neuroscience have increasingly examined how neural processes align across individuals. Such alignment can emerge when people interact directly, communicate sequentially, or experience the same stimulus (Dumas & Fairhurst, 2021; Hasson & Frith, 2016; Nummenmaa et al., 2018). This phenomenon is commonly referred to as interbrain synchrony (IBS), or interpersonal neural synchrony (INS), and describes the temporal coupling of neural signals between individuals (Hasson et al., 2012; Koban et al., 2019; Shamay-Tsoory et al., 2019). IBS has been associated with a wide range of social and cognitive processes and outcomes, including cooperation, coordination, and conflict (Czeszumski et al., 2022; Gamliel et al., 2021; Réveillé et al., 2024; Yang et al., 2020; Zhang et al., 2020a, 2023), learning (Kostorz et al., 2020, 2022; Zhang et al., 2024), developmental processes (Hoehl et al., 2025; Reindl et al., 2018), friendship and partnership (Ma & Liu, 2024; Parkinson et al., 2018; Zhao et al., 2024), and social impairments observed in psychiatric conditions (Konrad et al., 2024; Schilbach, 2016; Schilbach & Redcay, 2024). Synchrony has become one major concept of social connection (Baek et al., 2025).

IBS has been investigated across contexts, ranging from tightly controlled laboratory experiments to naturalistic and artistic environments (Dikker et al., 2021; Nummenmaa et al., 2018), using a wide variety of experimental paradigms. In shared-stimulus paradigms, multiple individuals experience the same stimulus (such as narratives or videos), allowing researchers to examine shared neural responses across participants (Finn et al., 2022; Hasson et al., 2012; Sonkusare et al., 2019). This design framework is used in both naturalistic neuroscience and social or relational neuroscience (De Felice et al., 2025). Related approaches examine sequential interactions, for example, when neural or behavioral responses from one participant are presented to another at a later time (Hasson et al., 2012). At the other end of the spectrum are real-time interaction paradigms, in which neural activity from two or more individuals is recorded simultaneously using hyperscanning (Montague et al., 2002). Studying interacting individuals in this way has become a central focus of social neuroscience (Hari & Kujala, 2009) and is often referred to as two-person or second-person neuroscience (Dumas et al., 2011; Redcay & Schilbach, 2019; Schilbach et al., 2013), with hyperscanning serving as a key methodological approach (Czeszumski et al., 2020; De Felice et al., 2025; Hakim et al., 2023; Hamilton, 2020). Building on this work, the framework of relational neuroscience has recently synthesized the field, characterizing the contexts in which interbrain coupling is studied along the two dimensions of interpersonal closeness and interactivity (De Felice et al., 2025). Within this framework, only hyperscanning allows the study of coupling arising from real-time reciprocal social exchange rather than from shared input alone. The field has grown substantially over the past decade, with a recent review documenting an increase in hyperscanning studies from 6 in 2012 to 46 in 2022 (Hakim et al., 2023).

IBS is now an established approach across major neuroimaging modalities: functional magnetic resonance imaging (fMRI), electroencephalography (EEG), and functional near-infrared spectroscopy (fNIRS) (Hakim et al., 2023; Lotter et al., 2023). Among these, fNIRS has emerged as the dominant choice in hyperscanning (Hakim et al., 2023) due to its portability and relative robustness to motion artifacts, which enable face-to-face and mobile recordings, and its adaptability to developmental research (Babiloni & Astolfi, 2014; Ferrari & Quaresima, 2012; Jones et al., 2025; Moffat et al., 2024; Nguyen et al., 2021b; Pinti et al., 2020).

This growth in scope, across settings, paradigms, and modalities, has outpaced methodological standardization. The diversity of experimental paradigms and analytical approaches has created a need for frameworks that enable researchers to quantify and interpret interbrain synchrony consistently and transparently (Hakim et al., 2023; Holroyd, 2022; Yücel et al., 2025).

Consider a typical hyperscanning design involving interacting pairs of participants and two conditions. In a *synchronous* condition, participant pairs engage in an activity expected to promote interpersonal alignment, such as cooperation or coordinated action. In an *independent* condition, participants perform a similar task without interacting or coordinating. Brain activity is recorded from both participants, and the central analytical goal is to derive a measure of IBS for each pair under each condition by comparing the neural time series within pairs, and to test whether synchrony is reliably greater during the synchronous than the independent condition across pairs.

In fNIRS hyperscanning, the standard approach for quantifying IBS is wavelet transform coherence (WTC). Following influential earlier studies (Cui et al., 2012), which adopted approaches developed in geophysics (Grinsted et al., 2004; Torrence & Compo, 1998), WTC has been widely implemented across the field, becoming the most prominent method (Hakim et al., 2023). In fMRI, WTC had been established slightly earlier as a tool for investigating dynamic functional connectivity (Chang & Glover, 2010). Rather than directly correlating the raw time series, WTC evaluates how strongly two signals are aligned in time–frequency space. This allows researchers to identify the time point at which synchrony emerges and the frequency bands in which it occurs.

A simplified example of a WTC-based analysis pipeline is illustrated in Figure 1. First, signals are acquired and preprocessed. This typically involves converting raw optical density measurements into changes in hemoglobin concentration and reducing physiological and systemic noise. Each signal is then decomposed into a time–frequency representation using the wavelet transform. WTC is computed by comparing these representations, yielding a map that quantifies the alignment of the two signals across time and frequency. Coherence values are subsequently aggregated within predefined frequency bands and experimental conditions to derive a final IBS metric, which is statistically compared across pairs. Although the discussion that follows is grounded in this specific example, the considerations raised apply equally to comparable sequentially recorded time series, not only to simultaneous recordings.

**Fig. 1.**
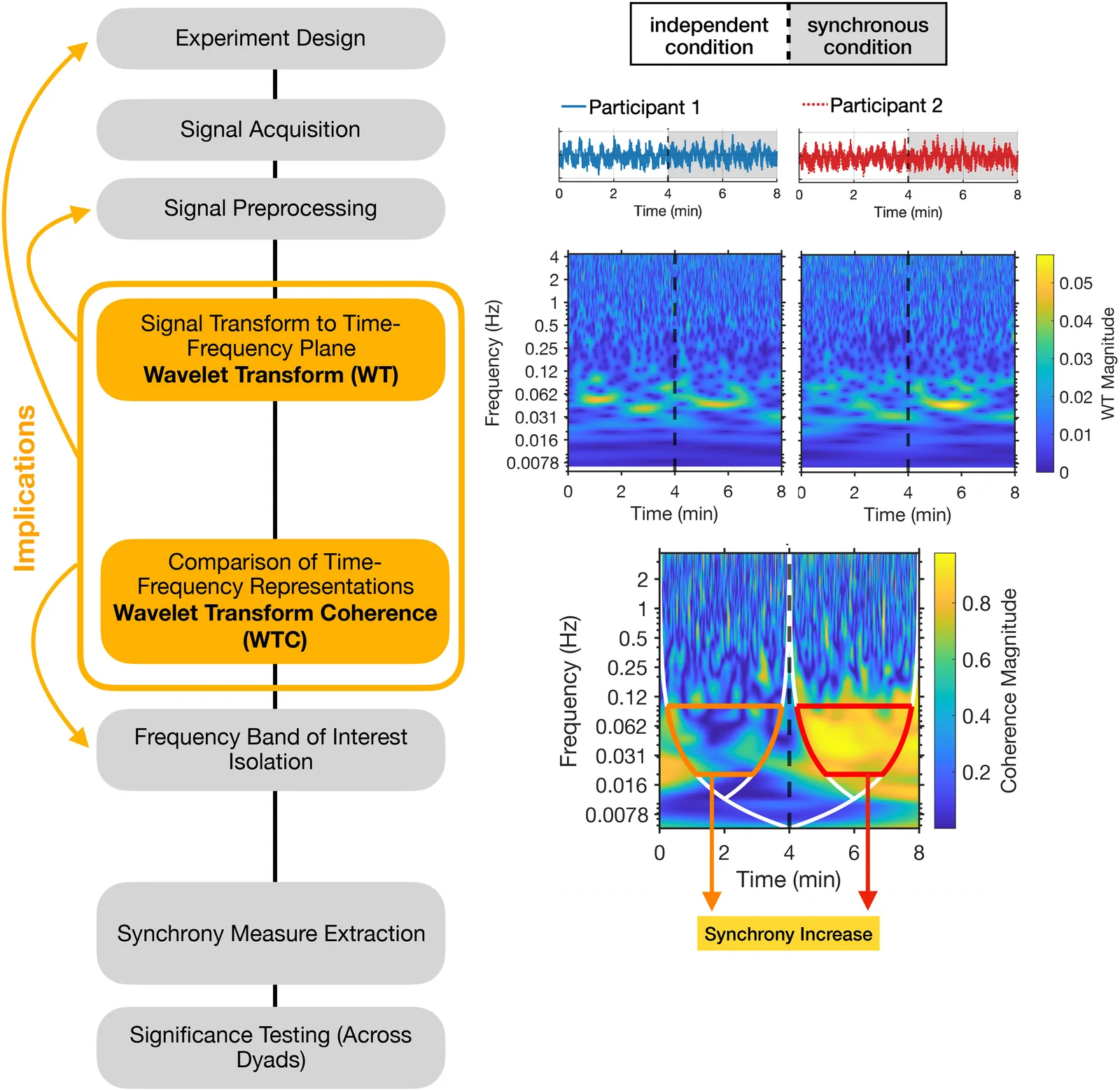
Example of a wavelet transform coherence (WTC)-based fNIRS hyperscanning analysis pipeline and a guide to the primer. Schematic overview of a typical analysis workflow from experimental design to statistical testing across dyads. The wavelet transform and wavelet transform coherence steps are highlighted in orange, indicating the primary focus of the first part of this primer. Arrows labeled “Implications” emphasize that methodological constraints of wavelet-based analysis affect multiple other stages of the pipeline, including design, preprocessing, and frequency band selection. The second part of the paper discusses these implications and derives practical recommendations for fNIRS hyperscanning research. The scalograms on the right correspond to simulated signals in a typical fNIRS frequency range.

The present primer focuses on two key steps in the IBS analysis pipeline (highlighted in orange in Fig. 1): the wavelet transform and the computation of wavelet coherence. Although these methods are widely used, their assumptions, constraints, and parameter choices are not always explicitly considered—particularly in how they interact with the properties of fNIRS data. Overlooking these factors can meaningfully influence experimental design and frequency-band selection, increase the risk of misinterpreting synchrony effects, and reduce reproducibility. At the same time, accessible methodological guidance remains limited: existing signal-processing literature is often highly technical and not tailored to the specific constraints of fNIRS or IBS research.

This primer aims to provide an intuitive, practically oriented introduction to wavelet-based methods for researchers entering the field of fNIRS hyperscanning or IBS, while also offering methodological insights for more experienced readers. To maintain accessibility, formal equations are largely omitted from the main text; readers seeking detail are referred to the Supplementary Material. Overall, the goal is to support informed decision making in experimental design, analysis pipeline construction, and the interpretation of IBS findings.

We proceed in two stages. In the first section, we introduce the foundations of time–frequency analysis, clarifying key concepts and developing an intuitive understanding of the time–frequency tradeoff before turning to wavelet-based representations. In the second section, we discuss these principles in the context of fNIRS signal characteristics, highlight caveats, and derive practical recommendations for different stages of the analysis pipeline.

## 2 Time–Frequency Analysis

### 2.1 Basic properties of oscillatory signals

In its simplest form, a signal is a sequence of measurements. In fNIRS, optodes emit near-infrared light and record changes in the intensity of light returning from tissue. The resulting time series of light-intensity measurements forms the recorded signal, from which changes in oxyhemoglobin and deoxyhemoglobin concentrations in the underlying tissue can be inferred (Scholkmann et al., 2014; Villringer & Chance, 1997).

In general, many signals exhibit oscillatory behavior, meaning they fluctuate around a baseline in a repeating manner. To introduce the terminology used to describe such signals, we consider the simplest example: the sine wave. It is periodic, meaning that the same pattern repeats exactly over time. One repetition of this pattern is called a cycle. A sine wave is fully characterized by its amplitude (A), frequency (f), and initial phase (φ_0_). The amplitude determines the magnitude of the oscillation, while the frequency specifies how rapidly it oscillates, that is, how many cycles occur per second. Its inverse, the period (P), is the duration of one complete cycle. The phase (φ) describes the signal’s position within the cycle at a given moment. It can be understood by representing one full cycle as a circle: as the signal progresses through a cycle, the phase advances around this circle from 0 to 360° (or 0 to 2π radians) before repeating. The initial phase specifies the starting point of the oscillation at t = 0.

These properties are illustrated in Figure 2, which compares pairs of sine waves that differ in one characteristic. Panel (a) shows waves that differ in amplitude. In panel (b), the waves share the same frequency but differ in phase, producing a constant shift of one waveform relative to the other. Panel (c) compares oscillations with different frequencies. Although the signals begin aligned, their peaks gradually separate because their cycles have different durations.

**Fig. 2.**
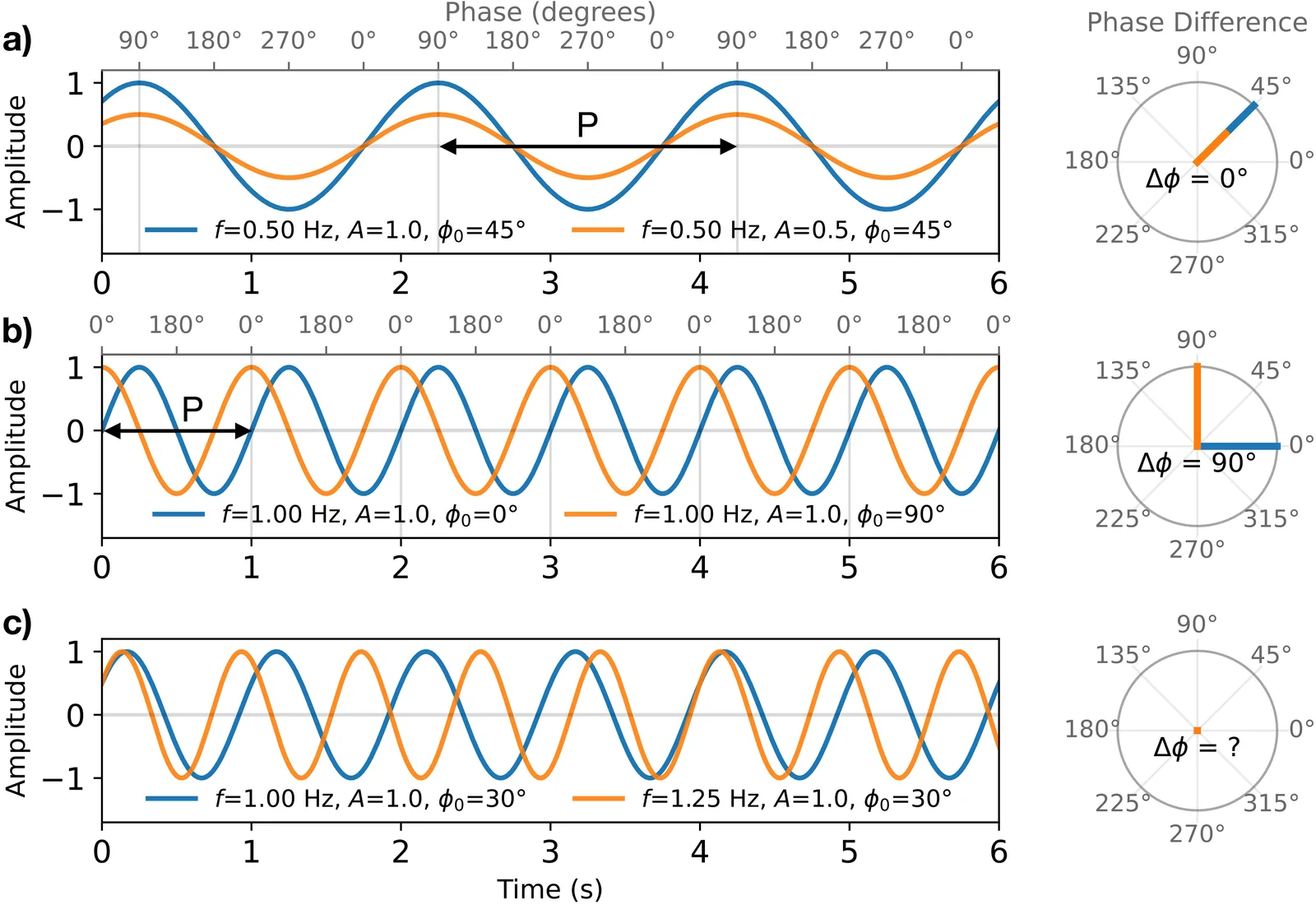
Basic characteristics of periodic signals in the time domain. The plots illustrate the fundamental properties of oscillatory signals: amplitude (A), frequency (f), period P, initial phase (φ_0_), and phase difference or offset. In examples (a) and (b), the lower x-axis denotes time, and the upper x-axis indicates phase (degrees); the time-domain signal is displayed on the left, and its corresponding phasor representation on the right, where the line length represents amplitude, and the angle represents initial phase. Adapted from Morales and Bowers (2022). (a) Two sine waves with equal frequency and phase but different amplitudes. (b) Two sine waves with equal amplitude and frequency but different initial phase (higher frequency, i.e., shorter period, than in a). (c) Two sine waves with equal amplitude and identical initial phase, but different frequencies. Their phase relationship changes continuously over time, so no single, well-defined phase difference can be assigned.

In this primer, synchrony refers to the temporal alignment between two signals. Two signals are considered synchronous when they oscillate at the same frequency and maintain a consistent phase difference Δφ over a given time interval, a condition often referred to as *phase locking*. This phase relationship specifies how the signals are aligned: signals are *in phase* when their peaks and troughs coincide (Fig. 2a), *anti-phase* when they are offset by half a cycle, and in a *lead–lag relationship* when one signal systematically precedes or follows the other by a fixed delay (Fig. 2b). Signals with different frequencies, as shown in Figure 2c, cannot remain synchronous because their phase relationship continuously changes, so any alignment between them is only transient. In contrast, differences in amplitude (Fig. 2a) do not affect synchrony, as they do not alter the temporal alignment between signals.

### 2.2 Three representations of a signal: Time, frequency, and time–frequency

The sine wave provides a useful starting point because its structure is simple: it oscillates with a single, well-defined frequency. Real physiological signals, however, such as fNIRS recordings, are far more complex. They contain multiple oscillatory components at various frequencies, whose strengths and presence may change over time. To illustrate how such composite signals can be represented, Figure 3 shows three complementary views of the same example signal.

**Fig. 3.**
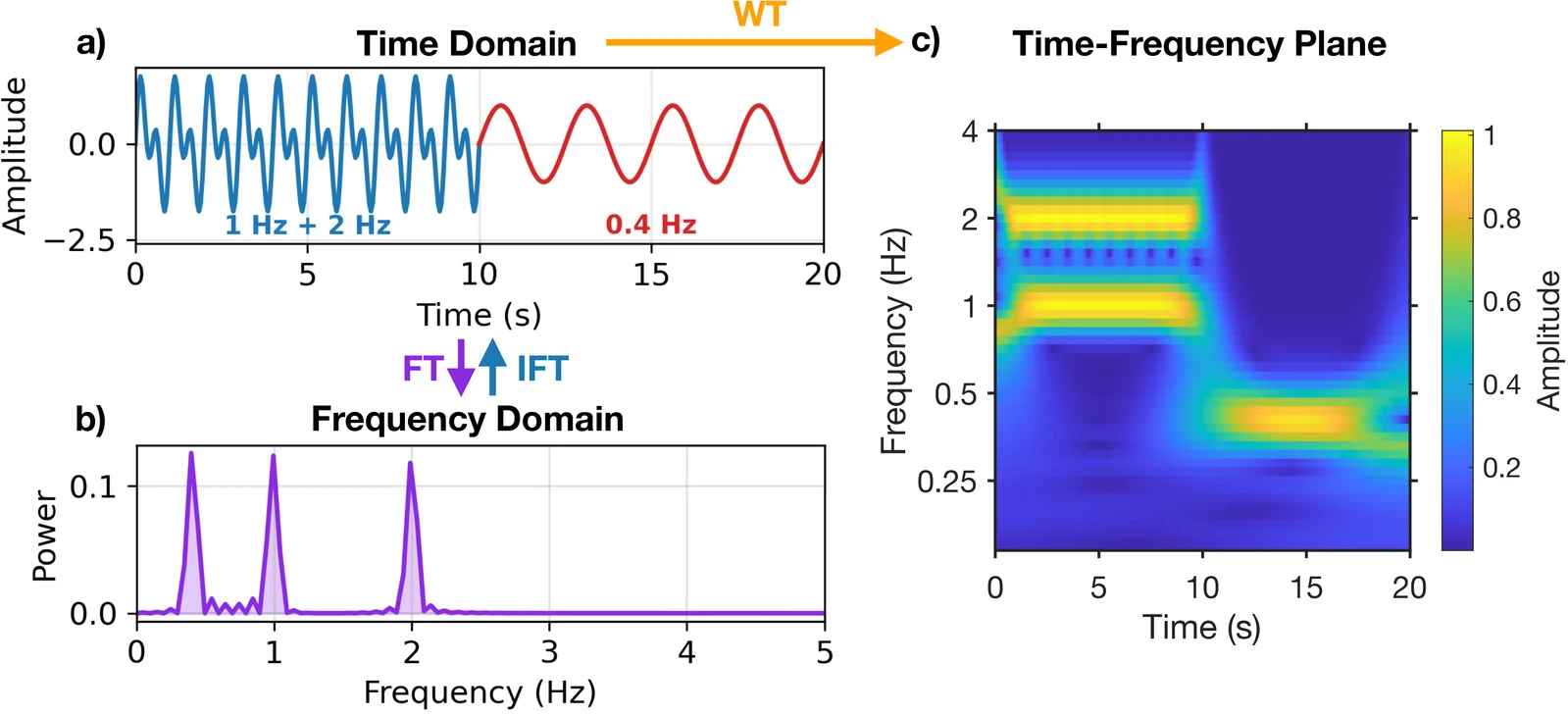
Three complementary representations of the same signal and the transformations between them. (a) Time-domain representation of a signal with changing frequency content. The first 10 seconds consist of higher-frequency oscillations (1 and 2 Hz added together), followed by a 10-second low-frequency oscillation. The time-domain view preserves when changes occur but does not explicitly identify which frequencies are present. The transformation from time domain to frequency domain was performed using the Fourier transform (FT), and the transformation to time–frequency space using the wavelet transform (WT). (b) Frequency-domain representation of the full signal detects the component frequencies as peaks in the spectrum. However, no information is provided about when in time these components are active. The peaks are not infinitely narrow, because each oscillation is present only for part of the recording; its frequency estimate has limited precision, resulting in a finite peak width. The FT yields two pieces of information: the strength (power = squared magnitude) of each frequency component (as shown here) and its phase (Supplementary Fig. S3). Reconstruction of the time-domain signal from the magnitude and phase spectra is achieved via the inverse Fourier transform (IFT). (c) Time–frequency representation of the signal obtained using the wavelet transform (WT). The scalogram decomposes the signal into its oscillatory components across time and frequency. The representation reveals when specific frequencies are active but exhibits spreading in both time and frequency. This apparent “smearing” reflects the intrinsic time–frequency trade-off and is a fundamental property rather than a methodological artifact.

When we imagine a signal, we usually think of its time-domain representation: how the measured value varies as a function of time. In the example in Figure 3a, the first half of the recording consists of higher-frequency oscillations, and the second of a lower-frequency oscillation. From the time-domain plot, it is clear when the change in oscillatory structure occurs at 10 seconds. However, the specific frequencies contributing to the recording are not immediately identifiable.

The same data can also be represented in the frequency domain using the Fourier transform (FT), which decomposes a waveform into its constituent frequencies. This reflects the general principle that any signal, including fNIRS recordings, can be decomposed into a set of simple sinusoidal components (see Supplementary Section S1 and Fig. S1). The result is a spectrum that describes how strongly different frequencies contribute to the signal. The spectrum contains two components: magnitude, which indicates the strength of each frequency (Fig. 3b), and phase, which describes how each frequency component is aligned in time (see Supplementary Section S1.3). Because the Fourier transform summarizes the entire recording, it reveals which frequencies are present overall but not when they occur.

Time- and frequency-domain representations contain exactly the same information, but organize it differently to emphasize different aspects of the signal. The classical FT allows us to examine either the signal’s timing or its frequency content, but not both simultaneously.

Time–frequency decomposition methods address this limitation by representing the signal jointly in time and frequency. The resulting time–frequency representation displays time on the horizontal axis, frequency on the vertical axis, and oscillatory strength as color. The representation in Figure 3c reveals that the higher-frequency components are confined to the first half of the recording, whereas the low-frequency component appears only in the second half.

However, this representation is not perfectly sharp. Although the signal was constructed from three well-defined sine waves (0.4, 1, and 2 Hz), the time–frequency plot does not show three narrow horizontal lines precisely localized in time and frequency (see also Supplementary Fig. S5). Instead, the components appear smeared: frequency bands are broad and temporal boundaries blurred. In other words, the estimates of which frequencies are present and when they occur are subject to uncertainty in both frequency and time. This uncertainty is not a flaw of the decomposition method but reflects a fundamental constraint of signal analysis, known as the time–frequency tradeoff.

### 2.3 The time–frequency tradeoff

The periodicity of a signal cannot be determined from a single instantaneous observation. Instead, it must be inferred from how the signal evolves over time. To identify a periodic or oscillatory pattern, the signal must be observed long enough to reveal the period of its behavior. To determine the frequency of a signal (that is, how many cycles occur per second), one must observe it long enough to capture at least one full cycle, and longer still to distinguish it from nearby frequencies.

To see why, consider two signals with slightly different frequencies. Over a short time window, their oscillations may appear nearly identical. However, as the observation window increases, small differences accumulate until one signal completes an additional cycle relative to the other. Only then does the frequency difference become unambiguous, and the two frequencies can be told apart.

Consequently, frequency estimation requires sufficiently long observation windows: the longer the signal is observed, the more precisely its frequency can be determined. (A formal yet intuitive derivation is provided in Supplementary Section S2.1.1 and Fig. S4.)

This principle has direct implications for time–frequency analysis. To estimate how frequency content evolves over time (as in Fig. 3c), the signal must be analyzed in finite segments, or windows, centered on each time point. The duration of this window determines the uncertainty in both time and frequency estimates.

A longer window contains more oscillatory cycles, improving frequency resolution: frequencies can be estimated more precisely and distinguished more reliably. However, this comes at the cost of temporal precision, as the exact timing of oscillatory components becomes less well defined.

Conversely, a shorter window improves temporal resolution, allowing changes in the signal to be localized more precisely in time. However, it contains fewer cycles, which increases frequency uncertainty and makes nearby frequencies harder to distinguish.

This relationship is known as the time–frequency trade-off: improving time precision necessarily reduces frequency precision, and vice versa. It is formalized by the Heisenberg–Gabor uncertainty principle (Gabor, 1946), which states that the product of temporal and frequency uncertainties is bounded from below (see Supplementary Section S2.1.1).

From this perspective, different signal representations occupy different positions along the tradeoff: time-domain representations emphasize temporal precision, frequency-domain representations emphasize frequency precision, and time–frequency representations balance the two, providing partial resolution in both domains.

As a result, time–frequency representations necessarily involve uncertainty and cannot achieve exact localization in both time and frequency. For fNIRS signals, this implies that inter-brain synchrony (IBS) cannot be resolved at arbitrarily fine temporal scales. Further fNIRS-specific considerations arising from this tradeoff are discussed in the second part of the manuscript.

### 2.4 Signal transform to the time–frequency plane

#### 2.4.1 Properties of the wavelet transform (WT)

Among the various time–frequency decomposition methods (Mallat, 1999), the wavelet transform (WT) has become the dominant approach in fNIRS analysis (Hakim et al., 2023). The WT compares a signal with a family of short oscillatory waveforms—*wavelets*. In this use case, a wavelet can be understood as a frequency detector: it is tuned to a specific frequency and reveals where in time, and how strongly, activity of that frequency is present in the signal.

The distinctive properties of wavelets become clearer when contrasted with sine waves (Fig. 4). A sine wave extends infinitely in time and contains infinitely many cycles. Its frequency is, therefore, precisely defined and appears as a sharp peak in the frequency domain. This property underlies the Fourier transform, in which sine waves serve as frequency detectors with high-frequency resolution but no temporal localization (Supplementary Fig. S2).

**Fig. 4.**
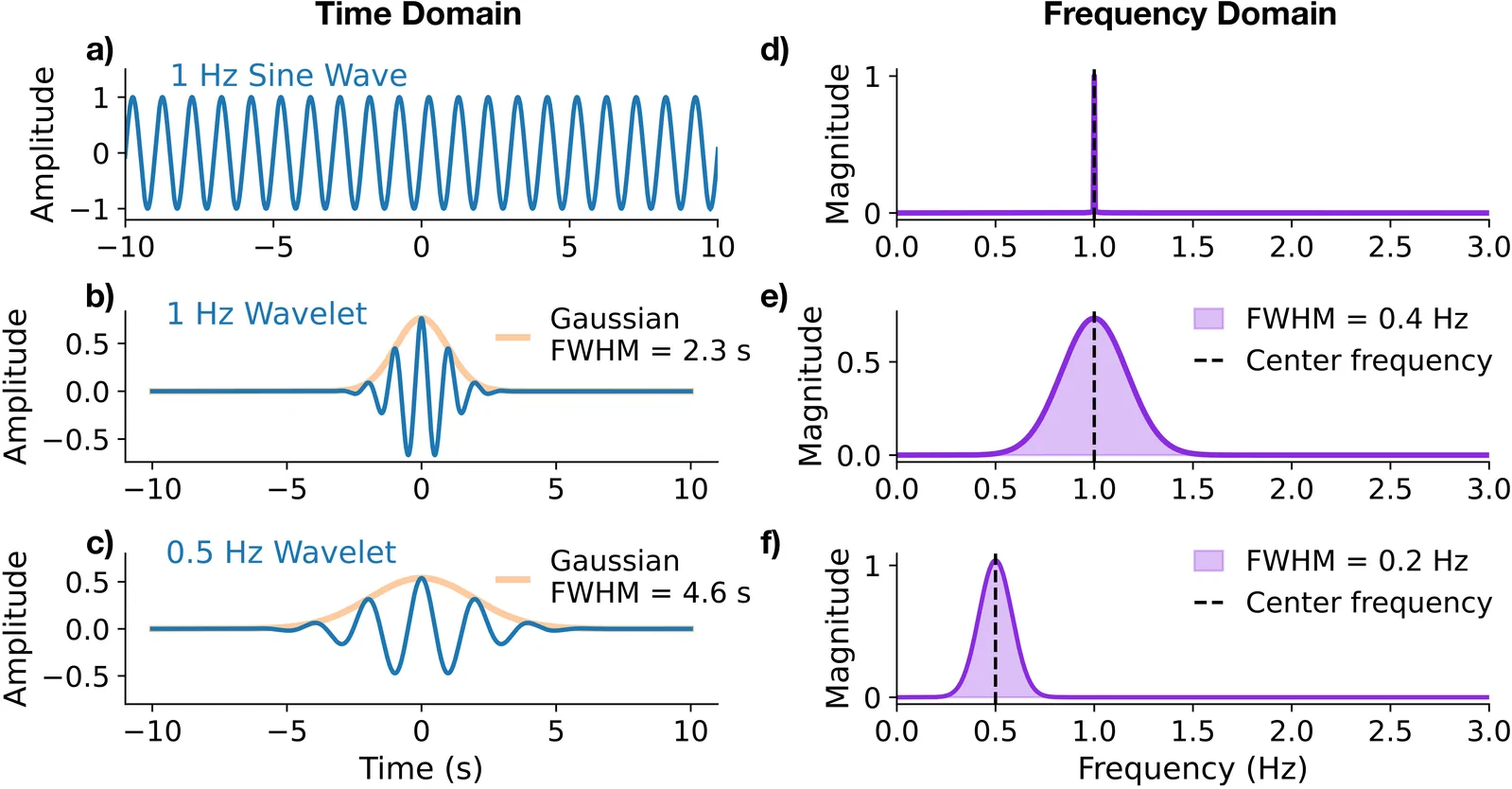
The sine wave and Morlet wavelet in the time domain and frequency domain. (a) Infinite 1 Hz sine wave. Because it extends indefinitely in time, its frequency is precisely defined. (b) 1 Hz real-valued Morlet wavelet in the time domain. The wavelet is constructed by shaping a sine wave with a Gaussian envelope, resulting in a localized oscillatory burst. The Gaussian envelope and its full width at half maximum (FWHM) are indicated. The Morlet parameter (here set to 6) controls the number of oscillations within the envelope and thus the time–frequency resolution (see Supplementary Fig. S10). (c) 0.5 Hz real-valued Morlet wavelet. Compared with the 1 Hz wavelet, it is stretched in time. (d) Frequency-domain representation of the sine wave shown in (a). (e) Frequency-domain representation (also called a spectrum or frequency response) of the Morlet wavelet shown in (b). The spectrum is approximately Gaussian, centered at the wavelet’s center frequency. The FWHM indicates the bandwidth. (f) Frequency-domain representation of the Morlet wavelet shown in (c). Compared with (e), the lower frequency exhibits a narrower frequency spectrum. In general, shorter wavelets have broader bandwidths, whereas longer wavelets have narrower bandwidths. When plotted on a logarithmic frequency axis, wavelets with different center frequencies exhibit approximately equal relative bandwidths (a constant ratio between bandwidth and center frequency) (Supplementary Fig. S8).

A wavelet, in contrast, is localized in time. The Morlet wavelet, commonly used for time–frequency decomposition, consists of a sine wave smoothly shaped by a Gaussian envelope. Its temporal resolution (that is, how precisely it can localize the timing of oscillatory activity) is determined by its temporal width. Its frequency resolution, that is, how well it can distinguish neighboring frequencies, is also limited, as it contains only a finite number of cycles. Rather than detecting a single exact frequency, a wavelet responds to a narrow range of neighboring frequencies around its center frequency; the extent of this frequency range is referred to as the wavelet’s bandwidth (Fig. 4e, f). The time–frequency tradeoff is, therefore, built directly into the wavelet itself (see Supplementary Section S3.5). Unlike the windowed Fourier transform, the WT provides a scale-adaptive relationship between wavelet width (i.e., temporal width) and the frequency range to be detected (see also Supplementary Sections S2.2 and S2.3).

Formally, the wavelet transform can be viewed as a pattern-matching procedure. This is illustrated in Figure 5. A basic waveform, called the mother wavelet, is selected. A family of related wavelets is then generated by scaling (stretching or compressing) this mother wavelet in time. Large scales produce long wavelets sensitive to slow oscillations; small scales produce short wavelets sensitive to faster fluctuations. At each scale, the wavelet is slid across the signal, and its overlap is computed to yield a wavelet coefficient at every time point (a process known as convolution). For complex wavelets, this coefficient is also complex, meaning it has both magnitude and phase: the magnitude reflects how strongly the signal expresses that pattern at that moment, while the phase encodes relative timing. Both are represented together in complex number Euler notation (see Supplementary Section S1.2.1).

**Fig. 5.**
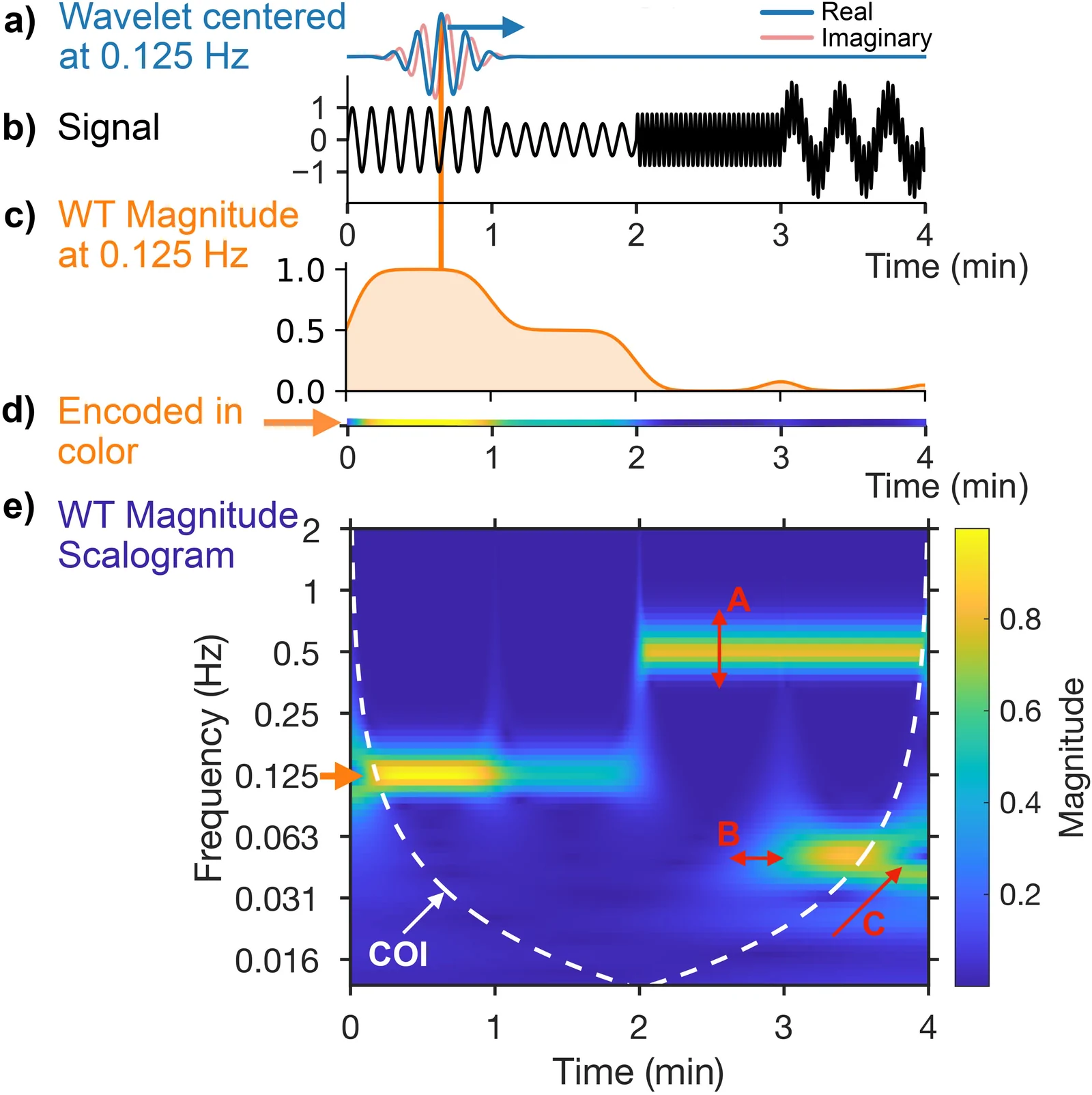
Construction and interpretation of the continuous wavelet transform (CWT). (a) Complex Morlet wavelet scaled so that it is centered at 0.125 Hz (real and imaginary components shown). (b) Example signal (4 minutes). Signal properties change every minute: minute 1: (0.125 Hz, amplitude 1); minute 2: (0.125 Hz, amplitude 0.5); minute 3: (0.5 Hz, amplitude 0.8); minute 4: (0.5 Hz, amplitude 0.8) and (0.05 Hz, amplitude 1). (c) Magnitude of wavelet transform coefficients obtained by sliding the 0.125 Hz wavelet across the signal. The upper plot shows magnitude as a function of time. The magnitude is L1-normalized and thus accurately reflects the amplitudes of the sine-wave components across frequencies (see Supplementary Section 3.4). (d) The same function as in (c) is displayed as a single colored row, where magnitude is encoded by color rather than by the y-axis. This row corresponds exactly to the 0.125 Hz row embedded within the full scalogram (panel e). (e) Wavelet scalogram. The row at 0.125 Hz (identical to the colored representation shown in panel d) is highlighted with an orange arrow. Red arrows indicate: (A) frequency uncertainty, (B) time uncertainty, and (C) edge effects. The cone of influence (COI) is marked by a white line; below it, border effects arising from the wavelet extending beyond the signal become non-negligible. Values outside the COI should, therefore, be interpreted with caution.

Repeating this procedure across multiple scales yields a scalogram: a two-dimensional representation that shows how oscillatory activity evolves over time and scale (i.e., a time–scale representation; Fig. 5e). When wavelets with well-defined center frequencies are used, as is the case for Morlet wavelets, each scale can be mapped to an equivalent frequency, allowing the scalogram to be expressed as a time–frequency representation. Here we focus on the complex Morlet wavelet, widely adopted for its favorable time–frequency concentration (Conraria & Soares, 2011) (see Supplementary Section S3). Time–frequency representations are sometimes broadly referred to as *spectrograms*; here, we use *scalogram* for wavelet-based representations and *spectrogram* for representations obtained using the short-time Fourier transform (see Supplementary Section 2.2).

Finally, to clarify terminology, this manuscript focuses on the continuous wavelet transform (CWT) and should be distinguished from the discrete wavelet transform (DWT). The CWT uses wavelets at continuous, highly redundant scales and time shifts, while the DWT uses a discrete set of scales and shifts. The CWT is typically used for exploratory time–frequency analysis and IBS analysis, whereas DWT-based methods are widely used in signal processing pipelines to identify or remove artifacts or nuisance signal components. For example, in fNIRS and EEG studies, DWT has been used for motion artifact correction (Brigadoi et al., 2014; Molavi & Dumont, 2012), ocular artifact removal (Krishnaveni et al., 2006; Phadikar et al., 2021), or denoising (Grobbelaar et al., 2022; Upadhyay et al., 2016). While DWT-based methods for artifact removal are based on similar signal-processing foundations, they are independent of the IBS analysis discussed in this manuscript and should, therefore, not be confused with it.

#### 2.4.2 Interpreting the scalogram

The scalogram in Figure 5e appears as a smooth, continuous image, with a coefficient defined at every time point and frequency. However, each coefficient reflects contributions from a wider neighborhood in both time and frequency. Because wavelets have finite bandwidth, adjacent frequency rows overlap substantially. Likewise, although coefficients are computed at every sampling point, each wavelet spans many samples in time. Consequently, neighboring rows and columns contain highly redundant information, and coefficients are not statistically independent.

The density of sampled frequencies is one of the parameters of the WT. Increasing it makes the image appear less pixelated. However, once adjacent wavelets substantially overlap in their frequency responses, increasing their density adds computational cost without revealing new details about the signal (Supplementary Fig. S5).

Because of their smooth and continuous structure, scalograms are particularly useful for qualitative inspection. Visual examination can help identify artifact-dominated channels and has been recommended as an fNIRS preprocessing step (Nguyen et al., 2021a).

The frequency axis is typically logarithmic rather than linear: successive frequencies differ by a constant ratio. This spacing arises naturally from the construction of the transform, in which wavelets are generated by stretching or compressing a mother wavelet by constant factors. As a result, although high-frequency wavelets have broader bandwidths in absolute (Hz) terms (Fig. 4), their relative bandwidth remains approximately constant, so on a logarithmic axis, wavelets at different scales appear to have equal bandwidth (Supplementary Fig. S8).

The labels in panel e of Figure 5 highlight several consequences of the time–frequency tradeoff. In (A), limited frequency resolution is reflected in the width of detected frequency bands. In (B), limited temporal resolution is most evident for low-frequency wavelets: because these wavelets are long in time, they extend across temporal boundaries, leading to apparent activity slightly before the true onset of a component. In (C), distortions appear near the edges of the recording. When a wavelet is centered close to a boundary, part of it extends beyond the available data. Most implementations address this by extending the signal (e.g., through reflection or zero-padding). As a result, coefficients near the edges are partly influenced by these artificial values rather than the observed signal and should, therefore, be interpreted with caution. The region where such effects become substantial is approximated by the cone of influence (COI) (Torrence & Compo, 1998), indicated by the white lines in Figure 5e. Its cone shape reflects the temporal extent of the wavelets: because low-frequency wavelets are longer in time, edge effects extend farther from the signal boundaries at lower than at higher frequencies. For the Morlet wavelet, the COI is defined using the e-folding time of its Gaussian envelope in time—that is, the time at which the wavelet’s power has decayed to 1/e^2^ (≈13.5%) of its maximum (Supplementary Figs. S9 and S11) (Torrence & Compo, 1998).

### 2.5 Comparison of time–frequency representations: Computation of synchrony

Having established how a single signal is represented in time–frequency space, we now extend this framework to compare two signals to determine their synchrony. The comparison is performed using the cross-wavelet transform (XWT) and its normalized form, wavelet transform coherence (WTC), which is the most commonly used metric for IBS analysis in fNIRS settings (Hakim et al., 2023).

#### 2.5.1 Cross-wavelet transform (XWT)

To compare two signals in time–frequency space, each is first decomposed using the wavelet transform. The cross-wavelet transform (XWT) then combines these representations pointwise, yielding a complex cross-spectrum. Its magnitude highlights regions where signals exhibit simultaneous activity at the same frequency, while its phase captures their relative timing, indicating which signal leads or lags.

The magnitude of the XWT depends on the amplitudes of the individual signals. A small XWT value at a given time–frequency point does not necessarily imply that the signals are unrelated: while it may indicate that they do not share an oscillatory component at that frequency and time, it may also reflect the presence of a shared component with relatively low amplitude. To quantify the strength of their relationship independently of amplitude, the cross-spectrum must be normalized. This is the purpose of the WTC, which captures the strength of the relationship between two signals without being confounded by amplitude.

This is illustrated in Figure 6. Panels (a) and (b) show the individual wavelet transforms of two synthetic signals. The XWT (panel c) highlights frequencies that are simultaneously present in both signals (1, 0.25, and 0.125 Hz). The 1 Hz band appears strongest because both signals have high amplitude there, whereas shared power is weaker at 0.25 Hz and faint at 0.125 Hz, where both have low amplitudes.

**Fig. 6.**
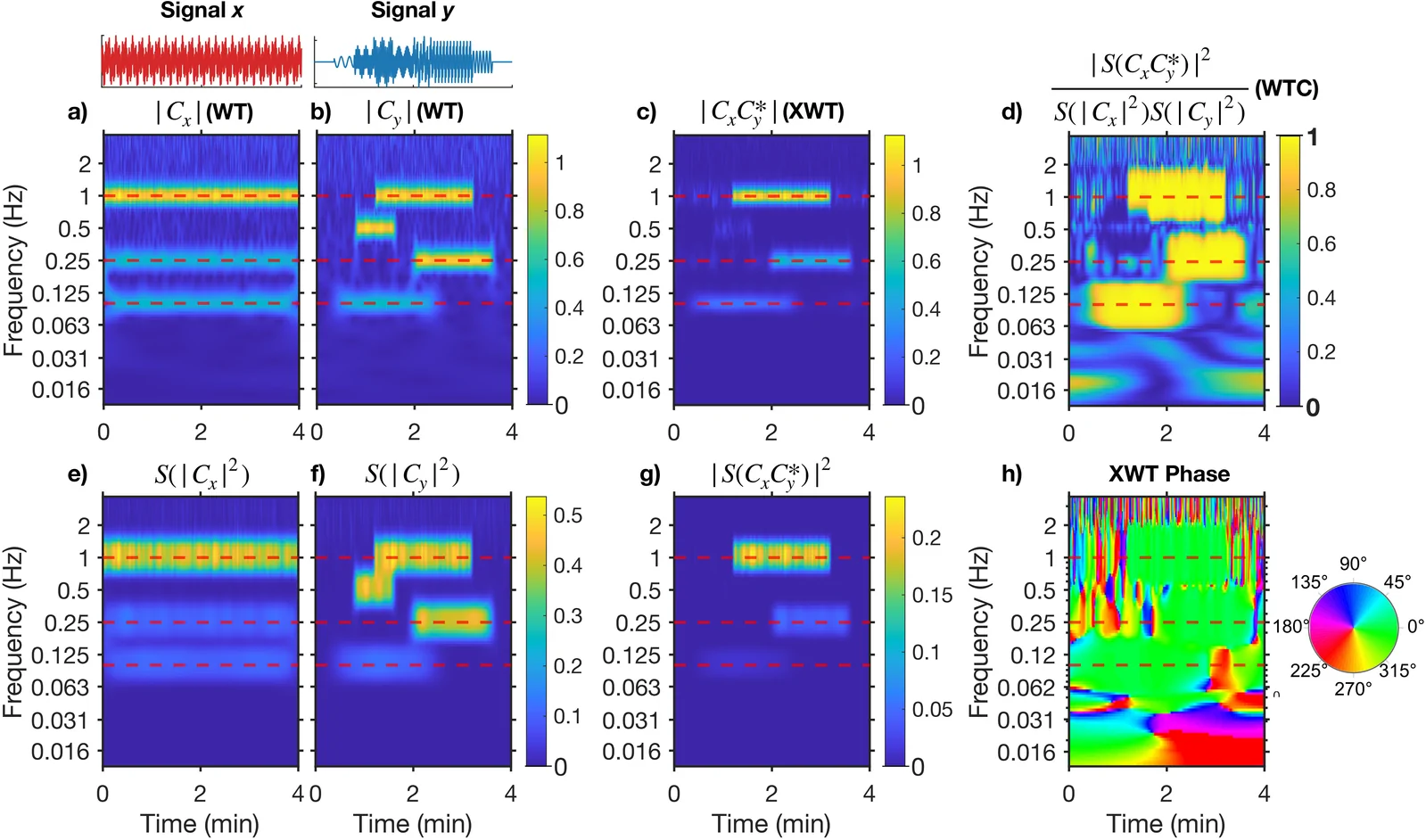
Cross-wavelet transform (XWT) and wavelet transform coherence (WTC) between two synthetic signals. Two signals, x and y, were constructed from sine waves at 1, 0.25, and 0.125 Hz, with added white noise (amplitude = 0.2). Signal x contains continuous oscillations, while signal y includes intermittent bursts at the same frequencies and an additional 0.5 Hz component. All components share the same initial phase (0°). (a, b) Continuous wavelet transforms (WTs) of x and y, shown as wavelet-coefficient magnitudes. The time-domain waveforms (noise removed for clarity) are shown above. (c) XWT, shown as cross-wavelet magnitude, highlighting regions where both signals contain power at the same frequencies. XWT magnitude scales with the product of the individual wavelet magnitudes (strongest at 1 Hz, weaker at 0.25 Hz, faint at 0.125 Hz). The 0.5 Hz component present only in y is absent. (d) WTC, a real valued measure bounded between 0 and 1. High coherence indicates oscillatory activity that is shared and phase-consistent over time, independent of amplitude. (e, f) Smoothed wavelet power of x and y. (g) Squared magnitude of the smoothed cross-spectrum. Panels (e–g) show the quantities used to compute WTC. (h) Phase of the smoothed cross-spectrum. Green regions (corresponding to high coherence) indicate a phase difference of 0° (signals in phase). Phase values in regions of low coherence should not be interpreted. Note that WTC (d) is normalized and, therefore, bounded between 0 and 1; the magnitude and power quantities shown in the other panels are not normalized, and their ranges depend on the amplitudes of the signal components.

#### 2.5.2 Wavelet transform coherence (WTC)

The WTC provides a normalized measure of how consistently two signals are related across time and frequency. It is conceptually similar to an absolute correlation coefficient: values near 1 indicate strong, stable relationships, while values near 0 indicate little or inconsistent coupling. Unlike a standard correlation coefficient, WTC estimates this relationship locally across the time–frequency plane. Because it is based on magnitudes, its values cannot be negative.

In practice, WTC is obtained by smoothing and normalizing the XWT. The normalization compares the smoothed cross-wavelet power (squared magnitude) with the smoothed power of the individual wavelet transforms. If this normalization were applied directly to unsmoothed scalograms, the resulting values would tend to be artificially high everywhere. Smoothing, therefore, plays a crucial role: it performs local averaging across neighboring time–frequency points, integrating information from a small region around each location in the time–frequency map (see Supplementary Section S4.1). This reduces noise and ensures that high coherence values reflect relationships that persist over time and frequency rather than momentary coincidences.

Panels (e–g) of Figure 6 show the quantities used in the coherence calculation: the smoothed individual wavelet power spectra (e–f) and the smoothed cross-wavelet spectrum (g). The coherence map (d) is obtained by dividing the squared magnitude of the smoothed cross-spectrum by the smoothed individual power spectra. This highlights the frequency bands where the signals maintain consistent relationships over time. Note that even though the example signals are composed of sine waves with well-defined frequencies, the regions of high coherence appear relatively broad. This reflects both the intrinsic time–frequency tradeoff of the wavelet transform and the additional smoothing required for coherence calculation.

#### 2.5.3 Phase relationships in wavelet transform coherence

In IBS analysis, researchers may ask not only whether two participants’ brain signals are synchronized but also whether one systematically precedes the other. For example, during coordinated action or turn taking, one partner may consistently lead. Such hypotheses concern not only shared frequency content but also relative timing. This is where phase information becomes relevant.

In the XWT, the relative phase indicates how the two signals are timed relative to each other at a given time and frequency. A phase difference of 0° indicates that the signals rise and fall together (in phase), whereas a difference of 180° indicates anti-phase behavior. Intermediate values reflect a systematic lead–lag relationship between the signals. The WTC itself is real valued and, therefore, has no phase of its own; phase relationships are typically derived from the smoothed XWT. In practice, phase differences are often visualized as arrows superimposed on the coherence map to indicate the direction of the lead–lag relationship (see Supplementary Fig. S13), or shown separately as a phase map (Fig. 6h).

Phase also plays a central role in the computation of coherence. In the smoothing step, neighboring time–frequency points of the XWT are averaged. This averaging incorporates both magnitude and phase information. When the phase difference between two signals remains stable across nearby times and frequencies, the contributions reinforce one another during smoothing, resulting in high coherence. If the phase relationship fluctuates or alternates, these contributions cancel out, and coherence is reduced (see Supplementary Fig. S12). High coherence, therefore, reflects not only shared frequency content but also a consistent temporal (i.e., phase) relationship between the signals.

Importantly, phase should only be interpreted in regions where coherence is present. When coherence magnitude is close to 0, the signals do not reliably share an oscillatory component at that time and frequency. Note that a phase of 0° is not “smaller” than a phase of 180°; phase describes relative timing, not strength. Without sufficient magnitude, the estimated phase difference is effectively random.

If phase information is to be used to infer lead–lag relationships between signals, it is important to remember that phase is defined relative to the oscillatory cycle rather than as an absolute time delay. The same phase offset, therefore, corresponds to different time shifts at different frequencies. For example, a phase lead of 90° represents a quarter of a cycle. At 0.1 Hz (period = 10 seconds), this corresponds to a lead of about 2.5 seconds, whereas at 0.02 Hz (period = 50 seconds), the same 90° offset corresponds to about 12.5 seconds. Thus, phase relationships are best interpreted within relatively narrow frequency ranges, where the corresponding time delays remain comparable. When coherence is averaged across broad frequency bands, phase differences may reflect very different absolute timing relationships.

Taken together, IBS, when operationalized through WTC, indicates that two brain signals are in synchrony if they are composed of the same frequency components and exhibit a consistent phase relationship. Lead–lag inferences from phase are meaningful only in regions where coherence magnitude is sufficiently strong and in narrow frequency bands.

## 3 Practical Implications and Recommendations for fNIRS Analyses

Having outlined the foundations of wavelet-based time–frequency analysis, we now turn to their implications for fNIRS IBS analyses. Coherence estimates depend not only on the analytical method but also on the properties of the measured signal. Therefore, we begin by considering the physiological and spectral characteristics of the fNIRS signal before deriving practical recommendations for experimental design and analysis.

### 3.1 Properties of the fNIRS signal

fNIRS measures changes in near-infrared light absorption by biological tissue from which relative changes in oxygenated (HbO) and deoxygenated (HbR) hemoglobin concentrations are derived (Ferrari & Quaresima, 2012). These hemoglobin signals do not reflect neural activity directly, but arise from neurovascular coupling—the physiological process linking neuronal activation to local changes in blood flow (Iadecola, 2017). The same mechanism underlies the blood-oxygenation-level–dependent (BOLD) signal in functional magnetic resonance imaging (fMRI) (Huppert et al., 2006). Owing to this close relationship, fNIRS and fMRI share key signal-processing characteristics and limitations.

In both modalities, neural activity is inferred through a vascular response that unfolds over several seconds. The hemodynamic response function (HRF) typically peaks about 4–6 seconds after stimulus onset (Glover, 2011) and evolves slowly thereafter (Lindquist et al., 2009; Miezin et al., 2000). Functionally, this response acts as a low-pass filter (Sauvage et al., 2017): rapid neural fluctuations are strongly attenuated, and only slower changes in activity (frequencies below ~0.3 Hz) are preserved in the measured signal. Although fNIRS is often sampled at higher rates than fMRI, the temporal information that can be meaningfully interpreted is constrained by these sluggish vascular dynamics.

However, the hemodynamic component of interest constitutes only one part of the measured signal, as the fNIRS time series also includes instrumental, systemic, and physiological noise (Scholkmann et al., 2014; Tachtsidis & Scholkmann, 2016). In studies investigating IBS, this distinction is crucial. When estimating synchrony, the goal is to quantify coupling between the hemodynamic components of the signals. However, wavelet coherence (and similar measures) detect any shared oscillatory activity, regardless of its origin. If non-neural processes become aligned across participants, synchrony estimates can be artificially inflated. For example, respiration or slow systemic fluctuations have been shown to synchronize during social interaction (De Felice et al., 2025). The central analytical challenge for IBS is, therefore, to isolate coupling in the hemodynamic component while minimizing the contribution of shared physiological and motion-related artifacts.

Several artifact sources occupy well-characterized frequency ranges (Reddy et al., 2021). Cardiac pulsation typically appears around ~1 Hz, respiration around ~0.2–0.3 Hz, and so-called Mayer waves or low-frequency oscillations (LFOs) around ~0.1 Hz (Scholkmann et al., 2014; Tachtsidis & Scholkmann, 2016). Even slower very low-frequency oscillations (VLFOs), often below ~0.01–0.05 Hz, may reflect systemic or instrumental drifts. Cardiac and respiratory components are usually well separated from the expected hemodynamic signal and can often be attenuated with appropriate preprocessing. In contrast, LFOs and parts of the VLFO range overlap with the frequency range in which task-evoked hemodynamic responses unfold (Kirilina et al., 2013; Yücel et al., 2016). These components are, therefore, more difficult to remove without also suppressing genuine neural signals.

### 3.2 Preprocessing: Filtering and artifact control

Filtering is widely used in fNIRS preprocessing to suppress physiological and motion-related artifacts (Pinti et al., 2019; Yücel et al., 2021). Typical implementations apply high-pass filters around ~0.01 Hz to remove slow drifts and low-pass filters between ~0.1 and 0.5 Hz to reduce cardiac and respiratory components (Pinti et al., 2019; St. Clair et al., 2025). The rationale is intuitive: remove frequencies unlikely to reflect neural activity and retain the slower hemodynamic fluctuations of interest.

Filtering is often treated as a routine preprocessing step, yet it fundamentally reshapes the signal. Any filter works by combining information across neighboring time points, effectively smoothing the data and blurring the precise timing of signal changes. This behavior reflects the same time–frequency trade-off that underlies wavelet analysis: increasing frequency selectivity necessarily reduces temporal precision.

A filter can be characterized by two related properties. Its *frequency response* describes which frequencies are retained or attenuated, while its *impulse response* shows how a brief, isolated event (a Dirac pulse) is spread over time by the filter. These two descriptions are intrinsically linked: sharper frequency cutoffs require longer impulse responses. A poorly parameterized bandpass filter can introduce temporal smearing that exceeds that of individual Morlet wavelets centered within the filtered frequency range, such that pre-filtering with sharp cutoffs may increase temporal uncertainty in the subsequent wavelet analysis.

Two further practical implications of filtering are particularly relevant for studies investigating IBS. First, filters can introduce temporal shifts. Depending on their phase characteristics, filters may delay the signal uniformly or shift different frequencies by different amounts (de Cheveigné & Nelken, 2019). If both participants’ signals are processed identically, their relative phase is preserved, although the signals themselves may be shifted in time. This makes it essential to apply the same preprocessing pipeline to both participants’ data. Nevertheless, temporal shifts must be accounted for when relating the timing of synchrony to the timing of experimental events.

Second, filters with narrow passbands or steep cutoffs can introduce artificial oscillations, often referred to as “ringing.” These oscillations were not present in the original signal but arise from enforcing abrupt frequency boundaries. Because wavelet coherence detects shared oscillatory structure regardless of origin, such filter-induced rhythms may appear as spurious synchrony. For a clear and accessible introduction to these filtering phenomena, the primer by de Cheveigné and Nelken (2019) is recommended.

Importantly, wavelet coherence already performs a frequency-selective analysis. Decomposing a signal with the wavelet transform can itself be understood as filtering: each wavelet isolates activity within a limited frequency range while integrating information over a corresponding time window. When coherence is computed within a predefined frequency band, and values are averaged across that band, this procedure effectively functions as a bandpass operation in the time–frequency domain. However, the Morlet wavelet’s frequency response is Gaussian shaped rather than sharp, meaning that frequencies outside the chosen band are gradually reduced, not abruptly removed. This gradual profile must be considered when defining frequency ranges, especially near known artifact bands, as synchronized artifacts may be “smeared in” due to the WTC’s frequency uncertainty (Fig. 7).

**Fig. 7.**
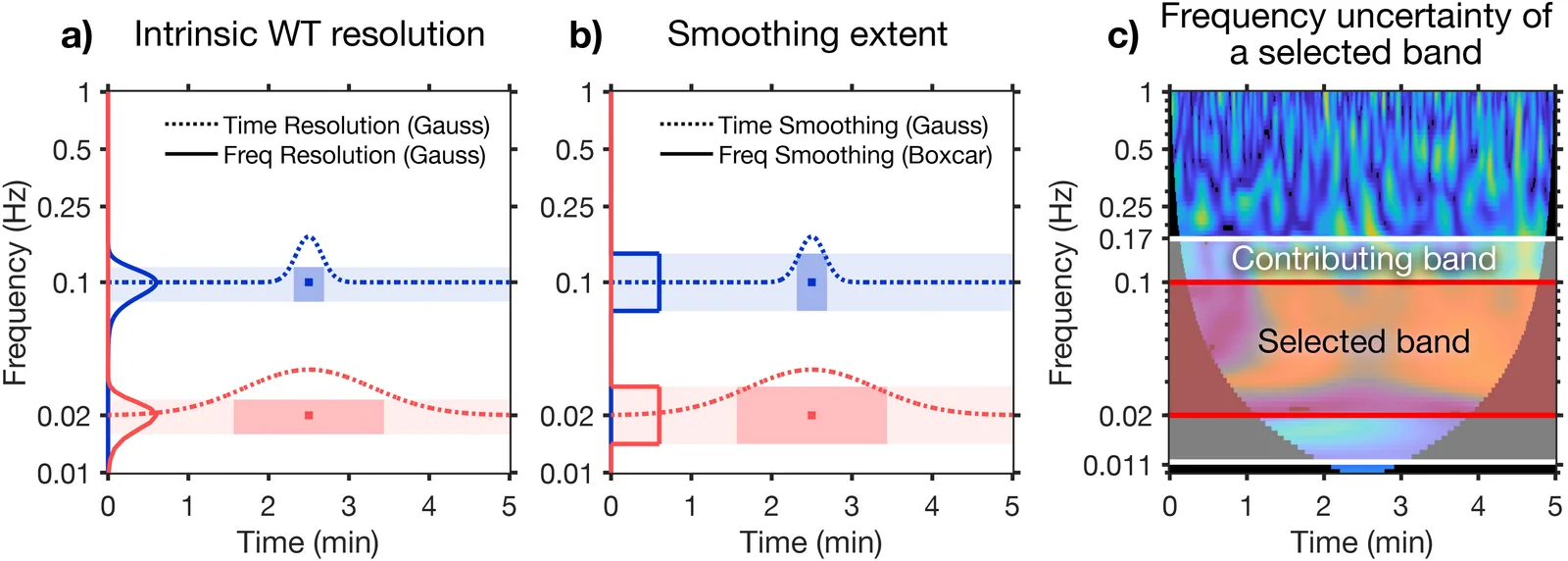
Effective time–frequency resolution of wavelet coherence. Three panels illustrate the intrinsic and smoothed time–frequency resolution of the wavelet transform coherence. (a) Intrinsic resolution of the continuous wavelet transform. For two example frequencies relevant to fNIRS hyperscanning (0.1 and 0.02 Hz), the frequency-domain bandwidth and temporal extent of the corresponding Morlet wavelet are shown. The shaded rectangles illustrate the joint time–frequency uncertainty of individual coefficients: in the calculation of the coefficient represented by the marker, significant contributions from the shaded time–frequency span are included. Rectangle dimensions correspond to the full width at half maximum (FWHM) of the Gaussian profiles. (b) Extent of smoothing applied during WTC computation. Frequency-domain smoothing is implemented across one octave, and temporal smoothing matches the Gaussian width of the corresponding wavelet. This expands the effective uncertainty beyond the intrinsic wavelet bandwidth. (c) Consequences for band selection. A band selected for analysis (0.02–0.1 Hz) is shown on a WTC scalogram. Due to intrinsic bandwidth and smoothing, the actual contributing frequencies extend beyond the selected band (indicated schematically by the wider “contributing band”, which is an estimate based on chosen bandwith and smoothing criteria, not a strict cutoff).

Because wavelet coherence already incorporates frequency selection, additional aggressive bandpass filtering may not be necessary in all cases. Instead, filters should be applied when clearly justified. When filters are used, their properties should be explicitly examined and reported. This includes inspecting both the frequency and the impulse response, as recommended by de Cheveigné and Nelken (2019). Potential ringing (i.e., spurious oscillations induced by sharp filter cutoffs) within the frequency band of interest should be checked.

Where possible, physiological artifacts should be recorded separately and removed using regression-based approaches rather than relying solely on frequency filtering (de Cheveigné & Nelken, 2019). Regression aims to estimate the unwanted signal separately and remove its contribution from the data. A common strategy is to include short-distance channels, which use closely spaced optodes and primarily capture superficial hemodynamics and artifacts from the scalp and extracerebral tissues rather than cortical activity. These signals can then be regressed from long-distance channels to reduce systemic contamination (Klein et al., 2022; Santosa et al., 2020; Wyser et al., 2020; Yücel et al., 2015). Motion artifacts, which often overlap with the low-frequency range of interest, are particularly difficult to remove via filtering. Recording motion with inertial measurement units (IMUs) and applying nuisance regression can reduce motion-related contamination, providing an alternative to filter-based approaches.

In summary, preprocessing decisions directly influence coherence estimates. Both participants’ signals must be processed identically, as filtering can introduce temporal shifts that bias synchrony. Because wavelet coherence already performs frequency-selective analysis, additional bandpass filtering should be applied only when justified and with careful inspection of filter properties. If possible, physiological and motion artifacts should be addressed through regression-based approaches rather than filtering alone.

### 3.3 Selection of frequency bands of interest

Selecting a frequency band of interest (FOI) for analysis requires balancing physiological plausibility with methodological constraints. Because the hemodynamic response restricts neural dynamics to slow fluctuations and several physiological artifacts occupy neighboring frequency ranges, interpretable neural synchrony is confined to a relatively narrow low-frequency window—typically around ~0.01–0.09 Hz (periods of roughly 11–100 seconds). Some people also consider higher frequencies (e.g., ≥0.2 Hz).

Coherence estimates near known artifact bands require particular caution due to the inherent uncertainty of frequency estimates in time–frequency methods. Isolating a frequency band on a scalogram does not impose a sharp spectral cutoff. Each wavelet coefficient reflects a range of neighboring frequencies, determined by the intrinsic bandwidth of the Morlet wavelet (Fig. 7a). In addition, the WTC involves smoothing across adjacent scales, often spanning approximately one doubling of frequency (i.e., one octave; Fig. 7b). Together, these factors broaden the effective frequency range contributing to each estimate. As a result, selecting a nominal band such as 0.02–0.1 Hz does not isolate only those frequencies; contributions extend beyond the specified boundaries (Fig. 7c). This is particularly relevant when analysis bands lie close to artifact-dominated regions, as neighboring frequencies may still influence coherence estimates. Consequently, apparent coherence within a selected band may partly reflect contributions from adjacent artifact frequencies. For this reason, artifact removal should preferably rely on independent artifact measurements, especially when the effective bandwidth of the WTC analysis overlaps with known artifact ranges.

Apart from time–frequency and artifact-related considerations, the FOI should also be defined a priori to avoid circular analysis (“double-dipping”). One approach is to predefine the band based on prior literature, theoretical considerations, or task structure. This preserves statistical independence and prevents biased inference. Although this may seem challenging in naturalistic paradigms, the physiologically meaningful frequency range in fNIRS is already constrained by artifacts and the low-pass characteristic of the hemodynamic response. This limits the search space and makes a priori specification more feasible than it may initially appear.

Alternatively, the FOI is sometimes determined empirically, for example, by identifying frequency bins that show increased coherence during a task relative to baseline (Zhou et al., 2026). However, this approach carries a substantial risk of circular analysis (Kriegeskorte et al., 2009; Zhou et al., 2026). If the FOI is selected using the same (or partially overlapping) data that are later used for hypothesis testing (e.g., by using the same experimental baseline twice, once for FOI determination and once for the main analysis), the analysis is no longer statistically independent. Selecting frequency bands based on elevated coherence within the same dataset introduces a positive bias and can lead to false positives.

To avoid this, FOI estimation must rely on data that are strictly independent of the primary analysis, for example, through dataset splitting, separate experimental runs, or independent participant samples. In addition, when the FOI is defined via bin-wise contrasts, statistical testing across individual frequency bins requires methods that account for dependencies between bins, along with appropriate correction for multiple comparisons. This dependency arises because time–frequency representations are redundant: adjacent frequency bins in the scalogram are not independent, as neighboring wavelets have overlapping frequency responses.

In practice, WTC is typically averaged across frequency bins within either a pre-specified FOI or a set of empirically identified significant bins, yielding a single coherence value per dyad. Although adjacent frequency bins are not independent, this dependence is of greater concern for bin-wise statistical testing than for inferential statistics across dyads: once coherence is averaged into a single per-dyad value, the within-dyad bin structure is collapsed, and the test across participants rests on the independence of dyads rather than bins.

### 3.4 Experimental design constraints

Once the FOI is defined, it directly determines the temporal resolution achievable in the experiment. In wavelet coherence, each estimate reflects signal information integrated over a finite time window whose width depends on the frequency under analysis. Changes in synchrony, therefore, cannot be localized more precisely than the intrinsic temporal extent of the selected frequency.

The cone of influence (COI) marks regions near temporal boundaries where wavelet coefficients are not fully supported by the data and must be excluded. In experimental designs, this applies not only to the beginning and end of the recording but also to every boundary between task conditions. To prevent coherence from leaking across adjacent blocks and biasing contrasts, the COI must be applied at each condition boundary (Fig. 8). This requirement determines the minimum block duration. Since the COI is defined by the e-folding time, a block must exceed twice the e-folding time of the lowest frequency in the FOI, once for each boundary. For example, at 0.02 Hz, the e-folding time for a Morlet wavelet is approximately 67 seconds, implying that blocks must be longer than roughly 2 minutes to yield usable coherence estimates. If blocks are shorter, most or all values at that frequency fall within the COI and cannot be cleanly attributed to the condition. Table 1 summarizes the temporal and spectral uncertainty of commonly analyzed frequencies and can guide experimental planning.

**Fig. 8.**
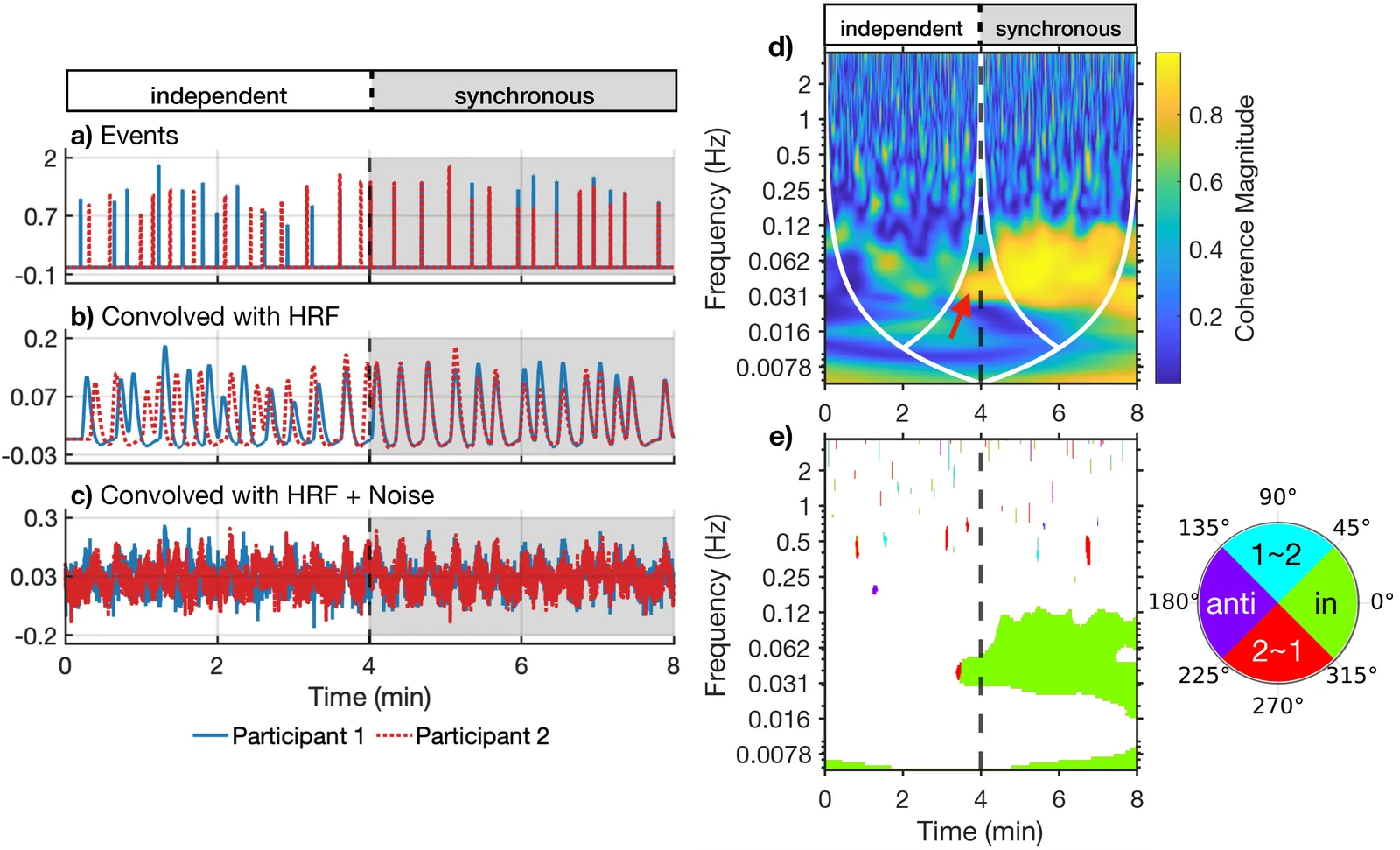
Simulated fNIRS signals and resulting wavelet coherence. Simulated event-related time courses were generated for two participants (one pair or dyad) under two conditions. In the independent condition, brief neural events (0.5 second duration) occurred randomly and independently in each participant (inter-event interval: 8–28 seconds; amplitudes drawn from a Gaussian distribution with mean 1 and standard deviation 0.2). In the synchronous condition, events occurred at the same time points for both participants, while amplitudes were sampled independently. (a) Event trains for both participants, separated into independent and synchronous conditions. (b) Event trains convolved with a canonical hemodynamic response function (HRF). (c) Addition of white noise. (d) Resulting wavelet transform coherence. Coherence is high in the synchronous condition and low in the independent condition. Notably, elevated coherence extends across the condition boundary (red arrow), despite synchronous events occurring only after the transition. Within each condition’s cone of influence (white lines), coherence values reflect the expected pattern. This illustrates that COIs must be considered separately for each block. (e) Phase of the cross-spectrum. Phase values are masked where coherence is below 0.7 (illustrative, arbitrary threshold; statistical significance should be empirically determined). Because events were temporally aligned, the phase difference is close to 0° (in phase) in coherent regions. In the phase legend, “1~2” indicates that Participant 1 leads Participant 2.

**Table 1.** Temporal and frequency-domain uncertainty measures of the complex Morlet wavelet (with Morlet parameter set to 6) at representative center frequencies relevant for fNIRS analysis.

Center frequency (Hz)	Center period (seconds)	$\sigma_{t}$ (seconds)	$FWHM_{t}$ (seconds)	e-folding time ≙ COI (seconds)	$\sigma_{f}$ (Hz)	$FWHM_{f}$ (Hz)
1.000	1.0	0.955	2.25	1.35	0.167	0.392
0.500	2.0	1.91	4.50	2.70	0.0833	0.196
0.200	5.0	4.77	11.2	6.75	0.0333	0.078
0.100	10.0	9.55	22.5	13.5	0.0167	0.039
0.075	13.3	12.7	30.0	18.0	0.0125	0.029
0.050	20.0	19.1	45.0	27.0	0.0083	0.020
0.025	40.0	38.2	89.9	54.0	0.0042	0.010
0.010	100.0	95.5	225	135	0.0017	0.0039

Note. σ denotes standard deviation and FWHM full width at half maximum.

This is also relevant to the investigation of dynamic, fluctuating IBS in naturalistic designs without fixed blocks and designs where falling in and out of synchrony is of interest (Gordon et al., 2025; Mayo & Gordon, 2020). Here, the same constraint applies: the minimal temporal precision of dynamic IBS estimates is effectively limited to twice the COI, as data near both ends of an analysis window must be excluded. Under current WTC practices, this often corresponds to temporal resolutions of 1 minute or longer (Kostorz et al., 2025).

Finally, particular attention should be paid to the choice of control conditions in hyperscanning study design. In practice, coherence in fNIRS hyperscanning is typically interpreted relative to a control condition that is either a resting condition or an active control. Depending on the research question, a good control condition should be closely aligned with the experimental condition, ideally keeping all ongoing processes comparable except for the effect to be isolated. In the case of hyperscanning, if particular social effects of the task are to be isolated, an active control that matches the task’s sensory and motor properties is substantially more appropriate than passive rest (Jones et al., 2025). When designing active control tasks, one should consider the experimental setting, perceptual input, somatomotor output, arousal levels, physiological confounders, cognitive processes that are independent of the effect of interest, and the temporal structure of the task at hand. Compared with passive rest, such controls provide a stronger basis for isolating interaction-specific coupling from shared task demands or physiological alignment. Further information about designing real-world fNIRS studies can also be found in Jones et al. (2025).

### 3.5 Statistical inference

WTC values range from 0 to 1 and can be positive even when the signals are unrelated; a WTC scalogram will typically contain regions of elevated coherence (Maraun & Kurths, 2004). The magnitude of coherence alone, therefore, does not establish whether observed coupling reflects interpersonal alignment of interest, co-variation driven by noise, or, depending on the hypothesis, alternative shared sources of co-variation (e.g., shared task structure). To address this, researchers typically evaluate observed coherence relative to both experimental control conditions, as described in the previous section, and null models.

For parametric testing of condition differences, generalized linear mixed models (GLMMs) should be used. Depending on the research question, GLMMs can account for participant-, dyad-, region-, and session-specific effects due to the nested, hierarchical data structure by modeling random effects. Ignoring the intrinsic hierarchy of the data and, for example, treating several measurement points of a dyad as independent may underestimate the uncertainty at hand. Additionally, WTC values are bounded, meaning they range between 0 and 1. A GLMM can account for non-Gaussian distributions. Often, differences in mean WTC across conditions are computed and Fisher *z*-transformed. As a more universal approach, WTC values may be modeled using a beta distribution (Nguyen et al., 2021a). Depending on the experimental design and research question, classical t-tests and ANOVAs may fail to account for the hierarchical structure and dependence of the data, and GLMMs are, therefore, strongly preferred.

Testing against a null model based on surrogate data methods provides a complementary approach. Surrogate data methods generate a null distribution via Monte Carlo simulation. First, a null hypothesis is specified (e.g., “the signals follow an autoregressive noise process”). Surrogate datasets are then generated to preserve the specific aspect of the null hypothesis while randomizing others. For each surrogate, the test measure (e.g., coherence) can then be obtained, and by repeating the procedure, its null distribution can be constructed against which empirical observations can be tested by counting how many surrogates are at least as extreme as the observed value. The resolution of the resulting p-value increases with the number of surrogate datasets; with *N* surrogates, the smallest attainable p-value is *1/(N+1)*, so a sufficient number of surrogates is required to ensure adequate statistical precision, particularly when correcting for multiple comparisons (e.g., across channels).

Surrogate data methods can be regarded as a nonparametric way to estimate a test statistic against a null hypothesis, or, informally, as a type of control experiment or benchmark (Theiler et al., 1992). While conceptually helpful and common, the latter analogy should not be taken too literally since the computationally generated surrogates are not statistically independent realizations of an experimental condition.

Available null-model implementations can be broadly divided into two families. Data-driven surrogates target intrinsic properties of the signals themselves; commonly used approaches include autoregressive and phase-scrambled methods (Conraria & Soares, 2011; Liégeois et al., 2021; Piazza et al., 2020; Reindl et al., 2018; St. Clair et al., 2025). In contrast, experiment-driven surrogates target pairing structure, typically by permuting participants across dyads (e.g., Nguyen et al., 2021a; St. Clair et al., 2025). A comprehensive overview of the construction of null models and the null hypotheses they test is provided by Lancaster et al. (2018).

Depending on the exact hypothesis at hand, the surrogates can be used to form an empirical null distribution of the chosen test statistic, to generate a reference summary statistic from the surrogate distribution (e.g., the mean; Nguyen et al., 2021a), or to create a null distribution of a GLMM parameter. In the latter, the null distribution of the parameter under test is obtained by fitting the GLMM to each surrogate dataset.

Random pairings, that is, a permutation of participants across dyads, are a common surrogate in hyperscanning. Random dyads and real dyads differ in the exact interaction scenario and potentially in task timing, but they share the same general experimental setting and tasks. A null hypothesis for a surrogate distribution could be: “The effect of the experimental condition on IBS arises to the same extent under random pairings of participants as under real pairings.” To test this, the condition coefficient is first estimated with a GLMM using the real dyads. Participants are then repeatedly re-paired at random, with each surrogate dataset containing the same number of dyads and preserving the original dataset’s relevant design structure. IBS is recomputed and the same GLMM is fitted to each surrogate dataset. Many such surrogate datasets are generated to obtain a sufficiently precise null distribution, against which the condition coefficient observed in the real dyads is compared. Depending on the subsequent hypotheses and tests used to examine IBS during social interaction, a significant contrast with random pairs may be a prerequisite for subsequent analyses.

Importantly, a significant difference between the null model and the observed WTC reflects only a difference in statistical properties under that specific null hypothesis, leaving other plausible explanations untested (Zhang, 2020c). Likewise, rejecting a null model does not, by itself, establish that IBS is of neurological or psychological relevance (Liégeois et al., 2021). That is a separate question, to be addressed through its relationship with behavior and through careful experimental design (Holroyd, 2022; Liégeois et al., 2021).

Taken together, several statistical approaches are used in WTC pipelines. A systematic comparison and further development of these methods, particularly for nonparametric testing and null models, are beyond the scope of this primer but remain important directions for future research.

### 3.6 Wavelet choice

Throughout this primer, we have illustrated wavelet-based analysis using the complex Morlet wavelet. However, the wavelet transform is a general framework, and different wavelet shapes can be used depending on which signal features one wishes to extract.

The Morlet wavelet is a common choice for time–frequency decomposition across scientific domains. Its Gaussian envelope gives it almost the smallest possible combined time–frequency spread allowed by the uncertainty principle (Conraria & Soares, 2011), meaning it can capture temporal and spectral detail simultaneously, nearly as precisely as theoretically possible (see Supplementary Section S3.5).

At the same time, fNIRS signals differ from signals that exhibit sustained oscillatory rhythms, such as EEG recordings, for which Morlet wavelets are commonly used (Cohen, 2019). Hemodynamic responses are typically transient and non-sinusoidal. In principle, alternative wavelet designs could, therefore, be used to better match these signal characteristics. For example, wavelets resembling the hemodynamic response function (HRF) have been proposed for hyperscanning applications (Zhang et al., 2020b), and specialized constructions such as “activelets” have been developed to capture hemodynamic activity by design (Khalidov et al., 2011).

However, such approaches introduce practical challenges. Wavelet analysis relies on stretching the mother wavelet across scales. Wavelets based on fixed temporal shapes (such as the HRF) may not generalize well across frequencies since the HRF itself does not scale across frequency bands. In addition, non-sinusoidal wavelets complicate the mapping between scale and conventional frequency measures, making frequency-domain interpretation less straightforward. At present, it remains unclear whether tailoring the wavelet shape substantially improves IBS analyses, and this remains an open methodological question.

### 3.7 Implementation and reproducibility considerations

Wavelet coherence analyses in fNIRS hyperscanning are most commonly conducted in MATLAB using toolbox functions. A common choice is the Cross-Wavelet and Wavelet Coherence Toolbox introduced by Grinsted et al. (2004) for geophysical time-series analyses. MATLAB now also provides a built-in Wavelet Toolbox (MathWorks, 2024) that closely resembles the original implementation and can be considered its further development. Python implementations have also begun to emerge; for example, the open-source HyPyP library, originally developed for inter-brain analyses in EEG and MEG, has recently been extended to support fNIRS studies (Ayrolles et al., 2021).

Regardless of the software environment, it is important to recognize that implementations often contain built-in methodological choices that can influence the resulting coherence estimates. These may include the selection of the mother wavelet, the width of smoothing in time and frequency, the handling of boundary effects, and normalization conventions. Such parameters are sometimes set implicitly by default values within a toolbox. To ensure comparability and reproducibility across studies, researchers should, therefore, inspect these settings carefully and report the key analysis parameters used in their pipeline.

### 3.8 Alternative measures of interbrain synchrony

This manuscript has focused primarily on WTC because it is currently the most widely used IBS metric in fNIRS hyperscanning. At the same time, it is important to view WTC as one member of a broader family of synchrony measures and to consider the present overview as a focused introduction rather than an exhaustive treatment of all available methods.

Wavelet analysis is attractive for its adaptive time–frequency resolution: lower frequencies are analyzed with longer effective windows and higher frequencies with shorter ones (Supplementary Fig. S7). This property is advantageous when signals span a broad spectral range, as in geophysical data (Grinsted et al., 2004; Jevrejeva et al., 2003), and in exploratory analyses where few prior assumptions about temporal or spectral structure can be made. In fNIRS hyperscanning, however, these practical advantages are less pronounced, as analyses are rarely fully exploratory and typically target a relatively narrow band of slow hemodynamic fluctuations, surrounded by artifact-heavy frequency bands.

In such cases, classical methods may offer conceptually similar alternatives. For example, the short-time Fourier transform estimates time-varying frequency content using fixed-length windows (Supplementary Fig. S6), and Fourier-based coherence applies a similar normalization to compare the resulting spectra of two signals (Cohen, 2014; Pachori, 2023). Hilbert-based approaches first apply band-pass filtering and then extract the instantaneous amplitude and phase of the filtered signal (Bruns, 2004). When signals are narrowband and parameters are chosen appropriately, these approaches often yield results similar to those obtained with wavelet-based methods (Bruns, 2004).

Windowed Pearson correlation, although less common in fNIRS IBS, is another intuitive measure of synchrony. It computes correlation within a sliding temporal window, producing a time-varying measure of coupling. Despite being less obvious, this approach also constitutes a time–frequency analysis: the finite window restricts the range of detectable frequencies and effectively imposes a low-pass constraint (Leonardi & Van De Ville, 2015). The spectral shape of the window can also introduce artifactual structure, potentially producing spurious correlations unless appropriately controlled, for example, through high-pass filtering during preprocessing (Leonardi & Van De Ville, 2015). Thus, windowed correlation should be interpreted with the same attention to the time–frequency trade-off and, in practice, requires similar choices of window length (temporal precision) as wavelet-based approaches.

Note that the results of a windowed Pearson correlation are not directly comparable with WTC results. A Pearson correlation coefficient ranges from −1 to 1 and depends on the exact value of the phase difference and the sign of the relationship between signals. Lagged correlation extends this approach by allowing one signal to be temporally shifted relative to the other, thereby identifying the delay that maximizes alignment. WTC, in contrast, ranges from 0 to 1 and quantifies the stability of coupling within a frequency band, largely independent of the exact phase difference, provided the phase relationship remains consistent over time. Consequently, the two methods capture different aspects of interdependence and may yield qualitatively different results, especially when signals share frequency content but differ in relative timing. These differences should be regarded as complementary rather than contradictory, and the chosen method should reflect whether the research question concerns signed amplitude co-fluctuations, delayed alignment, or frequency-specific, phase-stable coupling.

The narrowband characteristics of the fNIRS signal offer an advantage: phase-based methods could potentially be used. Phase synchrony is an established metric in EEG hyperscanning (Hakim et al., 2023) and an intuitive choice in this context, given the wavy nature of the EEG signal. Using a narrowband filter and the Hilbert transform, a phase signal could be extracted from the fNIRS signal as well. One could estimate intersubject phase synchrony in a manner analogous to approaches used in fMRI. Glerean et al. (2012) used a frequency band of 0.04–0.07 Hz to perform an intersubject phase synchrony analysis on fMRI data, a choice that would also be applicable to fNIRS data. A similar frequency band was used by Xu et al. (2020) for intersubject phase synchrony, and subdivisions of this range, as well as faster frequency bands, have also been used in fMRI analysis (Bolt et al., 2018).

While the WTC indirectly incorporates phase information, pure phase-based metrics such as phase-locking value or phase-lag index may yield insights into leader–follower relationships in dyadic interactions and thus provide information about the more fine-grained nature of the synchrony. Statistics are, however, harder to assess due to the circular nature of the phase, and are sensitive to preprocessing choices and differences in noise level (Bolt et al., 2018, Zimmermann et al., 2024). Phase values can also be extracted from the WTC calculations. However, given the current practice of averaging across frequency bands and time segments, careful methods are needed to correctly interpret the results. While we believe that this could be a promising metric for leader–follower dynamics, the complex nature of phase synchrony in non-stationary data warrants a separate investigation.

## 4 Conclusion

Our aim was to provide an accessible yet principled introduction to time–frequency analysis and wavelet transform coherence, equipping researchers with the solid foundations necessary for informed analytical decisions. To this end, we emphasized core principles rather than attempting an exhaustive exploration of the full parameter space, including alternative filters, preprocessing pipelines, or coherence metrics. Our focus was on clarifying the rationale behind commonly used methods, highlighting potential pitfalls, and providing practical guidance. The rapid expansion of fNIRS hyperscanning research underscores the importance of methodological transparency and convergence. As the field continues to grow, analytical choices are often adopted without explicit consideration of their assumptions. Making these assumptions visible is, therefore, essential for ensuring that findings remain interpretable and comparable across studies. Although framed in the context of fNIRS IBS analyses, the principles discussed here apply broadly to hemodynamic imaging and time–frequency methods more generally. Explicitly recognizing the constraints imposed by the time–frequency tradeoff is essential for robust experimental design, transparent reporting, and reliable interpretation of IBS findings.

## Supplementary Material

Supplementary Material

## Data Availability

All code used for this manuscript can be found at https://github.com/univiemops/fNIRS-Synchrony-Primer. No empirical data were collected; all data shown were generated via the provided code.
