# Supplementary material for: Interbrain synchrony analysis for fNIRS: A signal processing primer
Source: Imaging Neurosci (Camb). 2026 Oct 29;4:IMAG.a.1398. doi: 10.1162/IMAG.a.1398 (PMC13722052; doi:10.1162/IMAG.a.1398)
Supplement: Supplementary file 1 [file IMAG.a.1398_supp.pdf]

Supplementary Information

Interbrain synchrony analysis for fNIRS – a signal processing primer

Katarina Lodrant, Dimitri Van De Ville, Frank Scharnowski, Kathrin Kostorz

Table of Contents

S1 Foundations of Frequency Analysis 3

S1.1 Decomposition of signals into sinusoidal components 3

S1.2 The Fourier transform 4

S1.2.1 The Fourier transform as a template-matching procedure 4

S1.3 Frequency spectra 6

S2 Time-Frequency Analysis 8

S2.1 Time-Frequency Uncertainty 8

S2.1.1 The Heisenberg–Gabor uncertainty principle 8

S2.1.2 Time–frequency uncertainty in wavelet analysis 9

S2.2 The short-time Fourier transform 10

S2.3 Fixed vs adaptive time–frequency resolution 12

S3 The Morlet Wavelet and Its Properties 14

S3.1 Frequency-domain formulation 14

S3.1.1 Why start with a frequency-domain formulation? 14

S3.1.2 Wavelet spectrum in dimensionless frequency 15

S3.1.3 Wavelet spectrum in physical frequency (Hz) 16

S3.2 Mapping wavelet scale to frequency 17

S3.2.1 Fourier Factor (MATLAB definition) 17

S3.2.2 Fourier Wavelength (Torrence & Compo Definition) 18

S3.2.3 Comparison of the two definitions for $\omega 0 = 6$ 19

S3.3 Time-domain formulation 20

S3.4 Normalization conventions 22

S3.5 Time-frequency resolution 22

S3.5.1 Time-frequency tradeoff 23

S3.6 Practical measures of wavelet width 23

S3.6.1 Full Width at Half Maximum (FWHM) 23
S3.6.2 E-folding time 24
S3.6.3 Estimating temporal uncertainty for $\omega 0 = 6$ 25

S3.7 Constant-Q property 26

S3.8 The Morlet wavelet parameter $\omega 0$ 26

S3.9 The Cone of Influence (COI) 27

S4 Wavelet Transform Coherence 29

S4.1 Smoothing in wavelet coherence 29

S4.1.1 Smoothing in time 30
S4.1.2 Smoothing in frequency 30

S4.2 Role of phase in smoothing and the WTC 31

S4.3 Interpreting phase relationships 33

S1 Foundations of Frequency Analysis

S1.1 Decomposition of signals into sinusoidal components

Many signals encountered in scientific measurements contain oscillatory structure and can be understood as combinations of periodic components. The Fourier theorem states that any sufficiently well-behaved signal can be represented as the sum of sinusoidal waves that differ in frequency, amplitude, and phase. Even complex waveforms can therefore be viewed as the superposition of simpler oscillations.

Figure S1 illustrates this idea conceptually. A signal observed in the time domain is shown on the left. The central part of the figure represents the sinusoidal components that together form the signal. On the right, these components are expressed in the frequency domain, where each frequency component is characterized by a magnitude indicating its contribution to the signal and a phase angle specifying its temporal alignment relative to a reference oscillation.

This perspective provides an alternative way of describing a signal. Instead of characterizing it solely by how its amplitude changes over time, the signal can also be described by specifying the frequencies it contains and the magnitude and phase of each component.

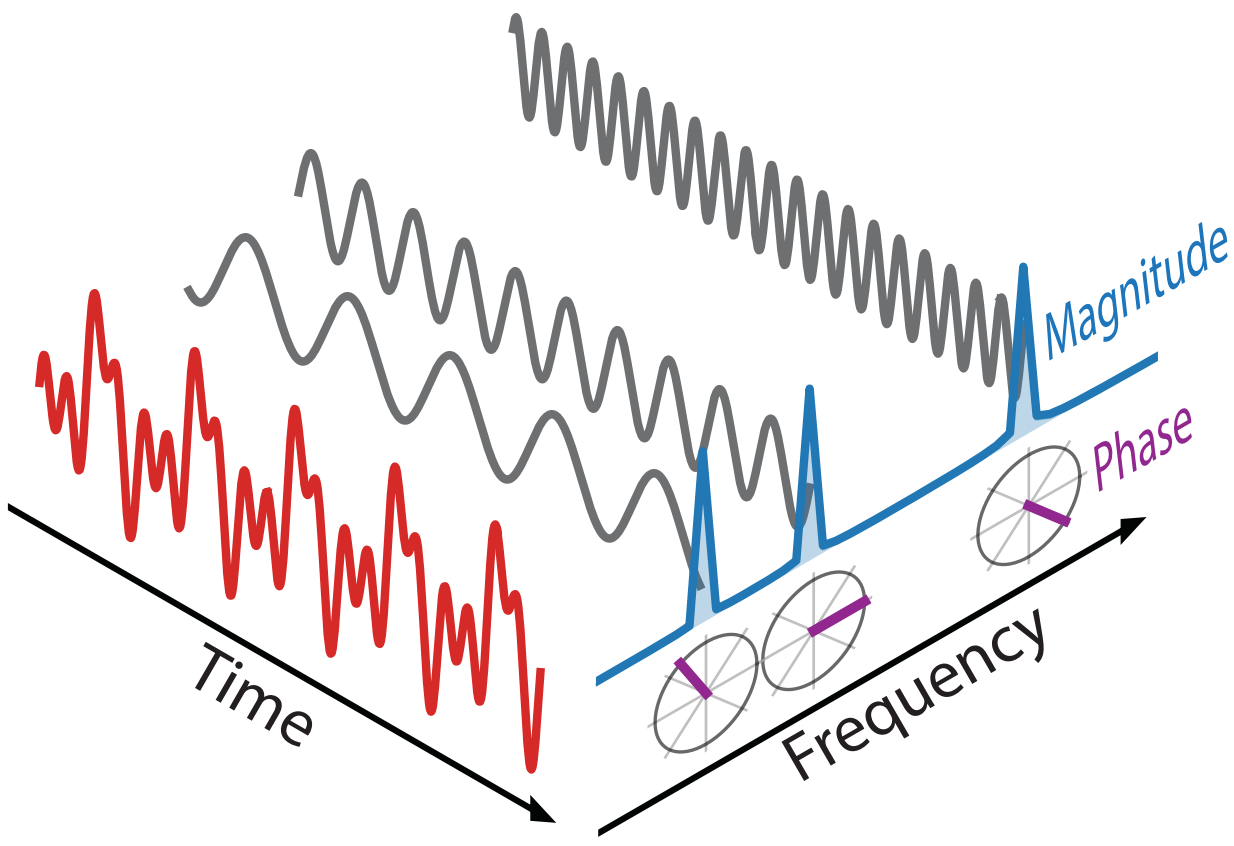

Figure S1. Time- and frequency-domain representations of a signal. *A signal observed in the time domain (red) can be interpreted as the sum of sinusoidal components with different frequencies (gray). The Fourier transform expresses this signal in the frequency domain by specifying the magnitude and phase of each component frequency (blue and polar plots). The magnitude indicates the strength of each frequency component, while the phase determines how these components align in time.*

S1.2 The Fourier transform

The Fourier transform provides the mathematical operation that converts a signal from its time-domain representation into its frequency-domain representation. Conceptually, it determines which sinusoidal components are present in the signal, along with their amplitudes and phases.

S1.2.1 The Fourier transform as a template-matching procedure

The discrete Fourier transform (DFT) can be understood as a systematic way of testing whether oscillations of different frequencies are present in a signal. To test for a component at a particular frequency, the signal is compared with sine and cosine waves at that frequency. This comparison is performed by multiplying the signal with the test wave point by point and summing the result over time.

If the signal contains an oscillation that aligns with the test wave, the product remains consistently positive or negative, and the resulting sum becomes large. If the signal does not contain that frequency, positive and negative contributions cancel out, and the sum remains close to zero. Repeating this procedure across all admissible frequencies yields the full frequency spectrum of the signal.

Figure S2 illustrates these principles. The signal is compared with sinusoidal test waves at different frequencies, and the resulting products are summed to quantify how strongly the signal resembles oscillations at those frequencies. Frequencies that are present in the signal produce large coefficients, while frequencies that are absent yield coefficients with magnitude close to zero.

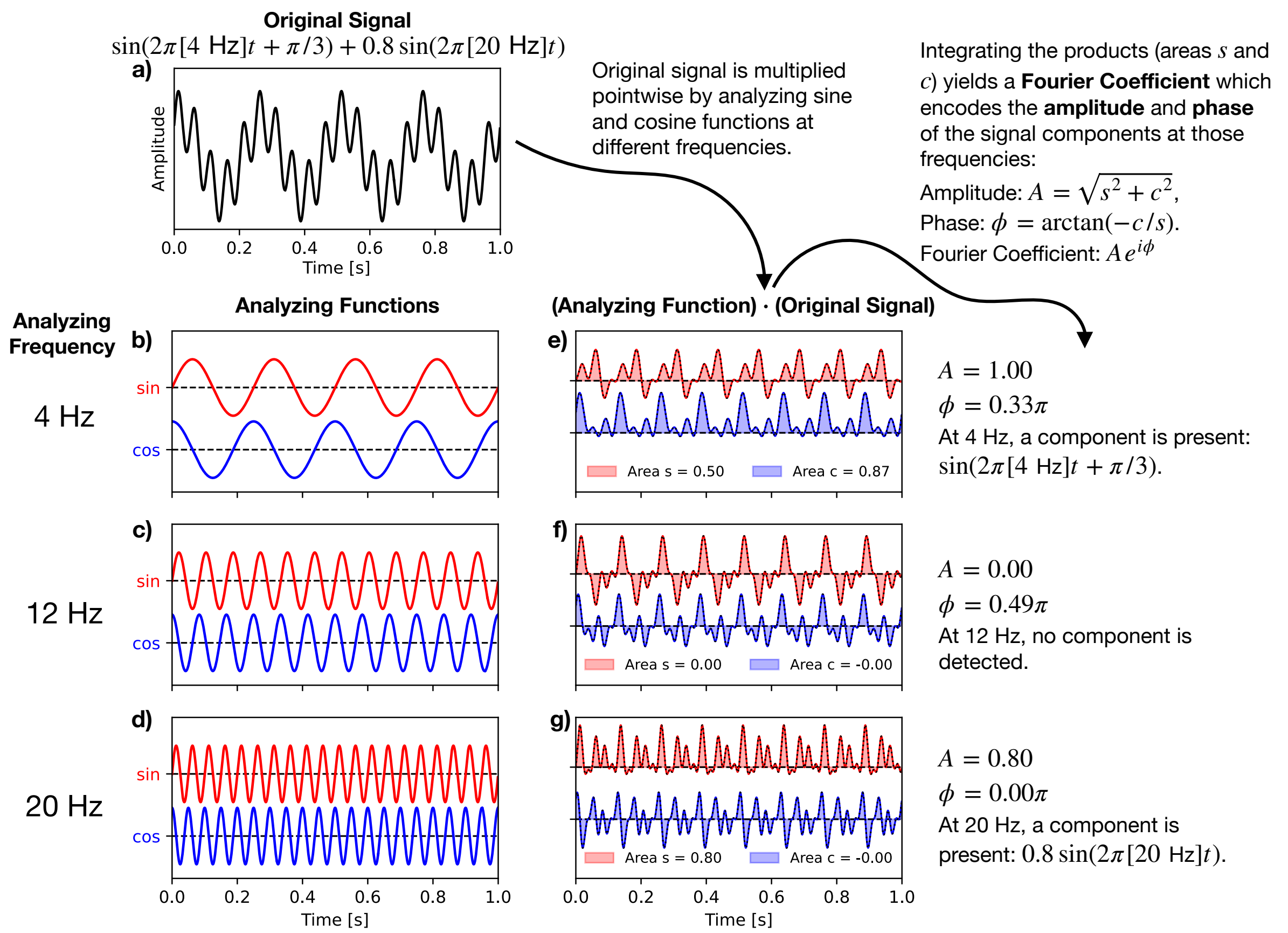

Figure S2. Demonstration of the Discrete Fourier Transform (DFT).

(a) The original signal is the sum of two sine waves with different amplitudes, frequencies, and initial phases (4 Hz with amplitude 1 and initial phase π/3, and 20 Hz with amplitude 0.8 and initial phase 0).

(b-d) Analyzing functions at three different frequencies: a pure cosine wave and a pure sine wave at each frequency.

(e–g) To test for the presence of a frequency component f, the signal is multiplied pointwise by an analyzing function (b-d). The integrals of these products yield the projection of the signal onto cosine and sine, from which the magnitude and phase at frequency f are determined. For f = 4 Hz and f = 20 Hz, the Fourier analysis recovers the exact amplitudes and phases of the components. For f = 12 Hz, no signal component is present, yielding a magnitude of zero.

This illustrates the principle of the Fourier transform: by sequentially projecting the signal onto sine and cosine waves at all candidate frequencies between the fundamental frequency (the inverse of the signal duration) and the Nyquist frequency (half the sampling frequency), one obtains the complete frequency spectrum.

In practice, the Fourier transform is often written using complex exponentials rather than separate sine and cosine waves. Euler's identity,

$$e^{ix} = cos(x) + i\, sin\,(x),$$

combines both functions into a single complex oscillation. In complex number notation, an oscillation with frequency *f* and amplitude *A* can therefore be described as

$$x(t) = Ae^{i2\pi ft}.$$

The transform therefore compares the signal with complex waves of the form $e^{i2\pi ft}$, producing complex Fourier coefficients that encode the magnitude and phase of each frequency component.

This interpretation highlights a conceptual link to the wavelet transform discussed in the main text. Both methods analyze a signal by comparing it to a family of oscillatory functions. In the Fourier transform, these functions are sine and cosine waves that extend infinitely in time and differ only in frequency. In contrast, the wavelet transform uses oscillatory functions that are localized in time and can be shifted and stretched to probe the signal at different times and scales. In both cases, the result is a set of complex coefficients that encode the magnitude and phase of the signal components.

S1.3 Frequency spectra

When a signal is represented in the frequency domain, the result is commonly referred to as its *spectrum*, which describes how different frequencies contribute to the signal. The Fourier transform returns complex coefficients for each frequency, containing both a magnitude and a phase. The magnitude indicates the strength of the oscillatory component at a given frequency, while the phase specifies its temporal alignment relative to a reference cosine wave.

Plotting the magnitude of the coefficients across frequencies yields the *magnitude* spectrum. After appropriate normalization, this is often referred to as the *amplitude* spectrum, as it reflects the amplitude of oscillatory components present in the signal. Squaring the magnitude produces the *power* spectrum, which describes how much of the signal's energy is associated with each frequency. Magnitude, amplitude, and power spectra therefore convey closely related information on the strength of frequency components.

The *phase* spectrum displays the phase angle associated with each frequency component relative to a cosine reference. Although less intuitive than magnitude, phase is essential for determining the temporal structure of a signal: two signals may have identical magnitude (or power) spectra yet differ in the time domain if their phase spectra differ.

Figure S3 illustrates these concepts. The left side shows a signal composed of two oscillatory components, and the right panels display the corresponding magnitude and phase spectra. Peaks in the magnitude spectrum correspond to the component frequencies, and the phase spectrum specifies their phase angles.

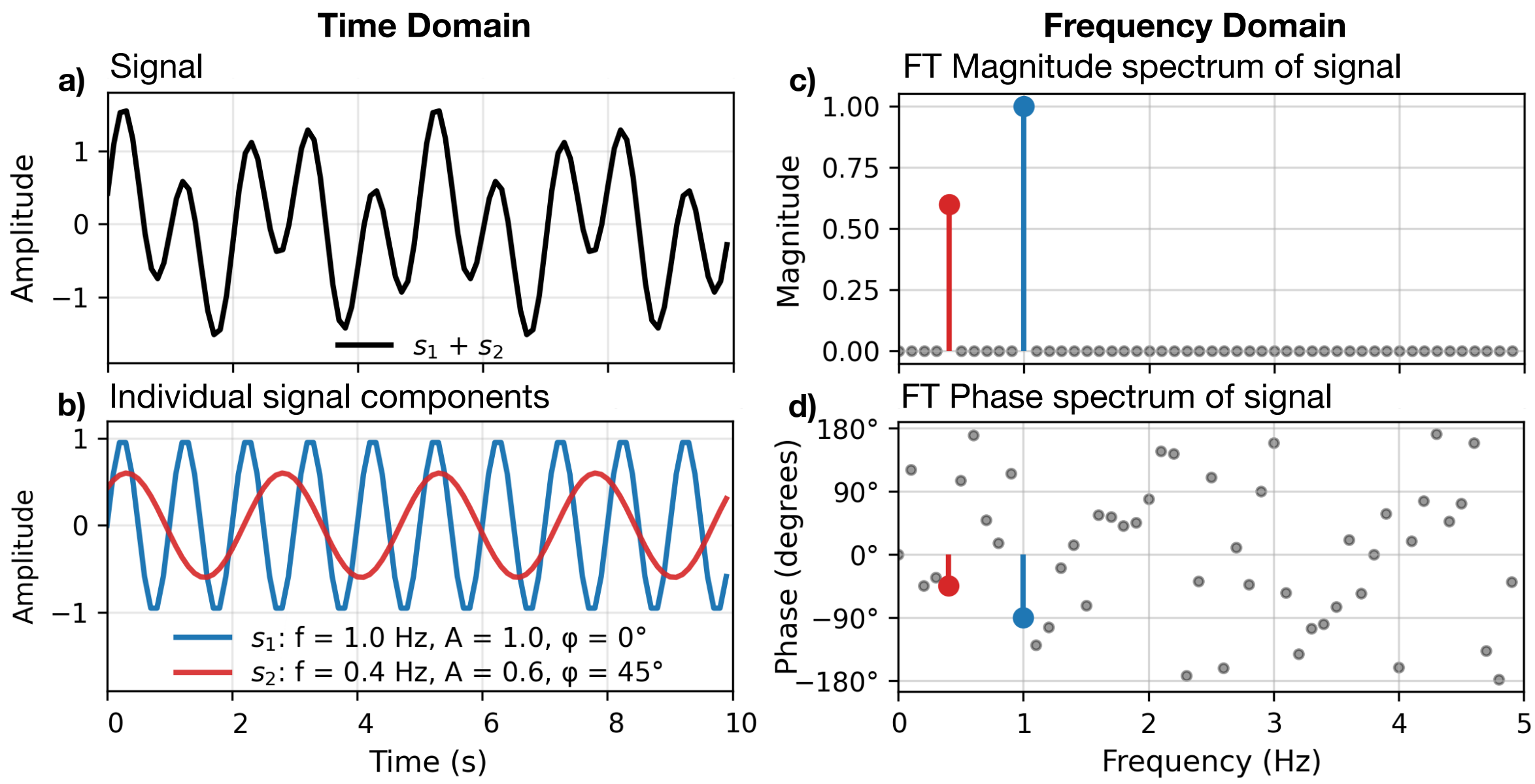

Figure S3: Time- and frequency-domain representations of a signal.

(a) The compound signal obtained by summing the components in (b).

(b) Two individual oscillatory components.

(c) Magnitude spectrum obtained via the Fourier transform (FT), showing peaks at the component frequencies.

(d) Corresponding phase spectrum, displaying the phase angles of each frequency component relative to a cosine reference.

Colored markers indicate frequencies with substantial magnitude; gray markers denote frequencies with negligible magnitude.

Phase values are returned by the Fourier transform for a range of frequencies, but they are only meaningful where the magnitude is sufficiently large. When the magnitude at some frequency is close to zero, the corresponding phase is effectively arbitrary because the Fourier coefficient is dominated by numerical noise. Phase interpretation should therefore be restricted to frequencies where the magnitude indicates a substantial contribution to the signal.

Finally, the range of frequencies that can be meaningfully analyzed in a signal is limited by its duration and sampling rate. The lowest resolvable frequency corresponds to one full oscillation over the entire recording (one period then being equal to the signal's duration).

Oscillations slower than this would not complete a full cycle within the data and cannot be uniquely identified. At the upper bound, oscillations faster than half the sampling rate cannot be accurately resolved, a limit known as the Nyquist frequency. Intuitively, a 10 Hz oscillation completes ten cycles per second; if the signal is also sampled at 10 Hz, only one sample per cycle is recorded, which is insufficient to capture its shape.

S2 Time-Frequency Analysis

S2.1 Time-Frequency Uncertainty

S2.1.1 The Heisenberg–Gabor uncertainty principle

In the main text, the Heisenberg-Gabor uncertainty principle was introduced conceptually. Here, we derive it more formally following MacLennan (2017). The key idea is that frequency cannot be determined from a single moment in time. Instead, it must be estimated by observing how the signal evolves over a finite time interval. As a result, improving the precision of a frequency estimate requires observing the signal for longer, which increases uncertainty about the exact time at which that estimate applies.

One simple way to estimate frequency is by counting the number of oscillatory cycles observed within a time interval. If N_{peaks} cycles occur during a time window Δt, the frequency can be estimated as

$$f = N_{peaks}/\Delta t.$$

To distinguish whether two signals have different frequencies, the observation window must be long enough for the number of observed cycles to differ (Figure S4). Consider two signals x and y with frequencies f_x an f_y, where $f_y = f_x + \Delta f$. Over an observation window of duration Δt, the expected number of cycles is

$$N_x = f_x \Delta t$$

for signal $x,$ and

$$N_y = (f_x + \Delta f)\Delta t$$

for signal y.

The difference in the number of observed cycles is therefore:

$$|N_y - N_x| = \Delta f \, \Delta t.$$

To reliably distinguish the two frequencies, the cycle counts must differ by at least one:

$$|N_y - N_x| \geq 1.$$

Substituting the expression above gives

$$\Delta f\, \Delta t \geq 1.$$

This relation captures the essence of the Heisenberg–Gabor uncertainty principle (Gabor, 1946): the product of time and frequency uncertainties has a lower bound. While the exact constant depends on the derivation (MacLennan, 2017), the central insight remains the same—time and frequency cannot be localized with arbitrary precision simultaneously.

In the peak-counting example above, this implies that resolving a frequency difference Δf requires observing the signals for a minimum duration

$$\Delta t = 1/\Delta f.$$

Smaller frequency differences can only be detected by extending the observation window. In other words, the more precisely we wish to determine frequency, the longer the observation window must be. Conversely, restricting the analysis to short time intervals inevitably increases uncertainty in the frequency estimate.

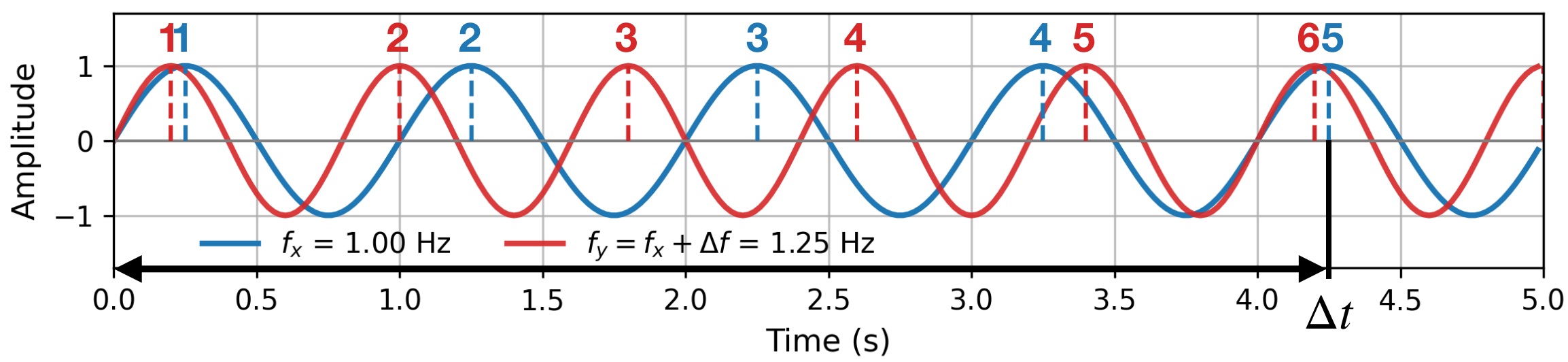

Figure S4. Illustration of the time–frequency resolution tradeoff.
Two sinusoids with identical amplitude and phase but slightly different frequencies. Initially, the signals remain aligned, but over time their cycles gradually drift apart. The minimum observation time required to distinguish the two frequencies corresponds to the interval Δt over which the signals' cycle counts differ by one. Adapted from MacLennan (2017).

S2.1.2 Time–frequency uncertainty in wavelet analysis

The time–frequency tradeoff derived above applies to all time–frequency decomposition methods, including the wavelet transform. Even when a signal contains oscillations with precisely defined frequencies and durations, the resulting time–frequency representation cannot localize these components with perfect precision.

Figure S5 illustrates this limitation. The example signal has a simple and well-defined time–frequency structure, yet the wavelet scalogram shows components that are spread in both time and frequency rather than sharply localized. This apparent "smearing" is not caused by

noise or poor method performance but reflects the intrinsic uncertainty of time–frequency representations in physics and signal processing. While one might intuitively expect to see Fig. S5a, in reality, only representations such as Fig. S5b-c are obtainable.

Increasing the density of frequency sampling does not overcome this limitation. While a scalogram computed with more frequency bins may appear smoother, the underlying time–frequency resolution remains unchanged.

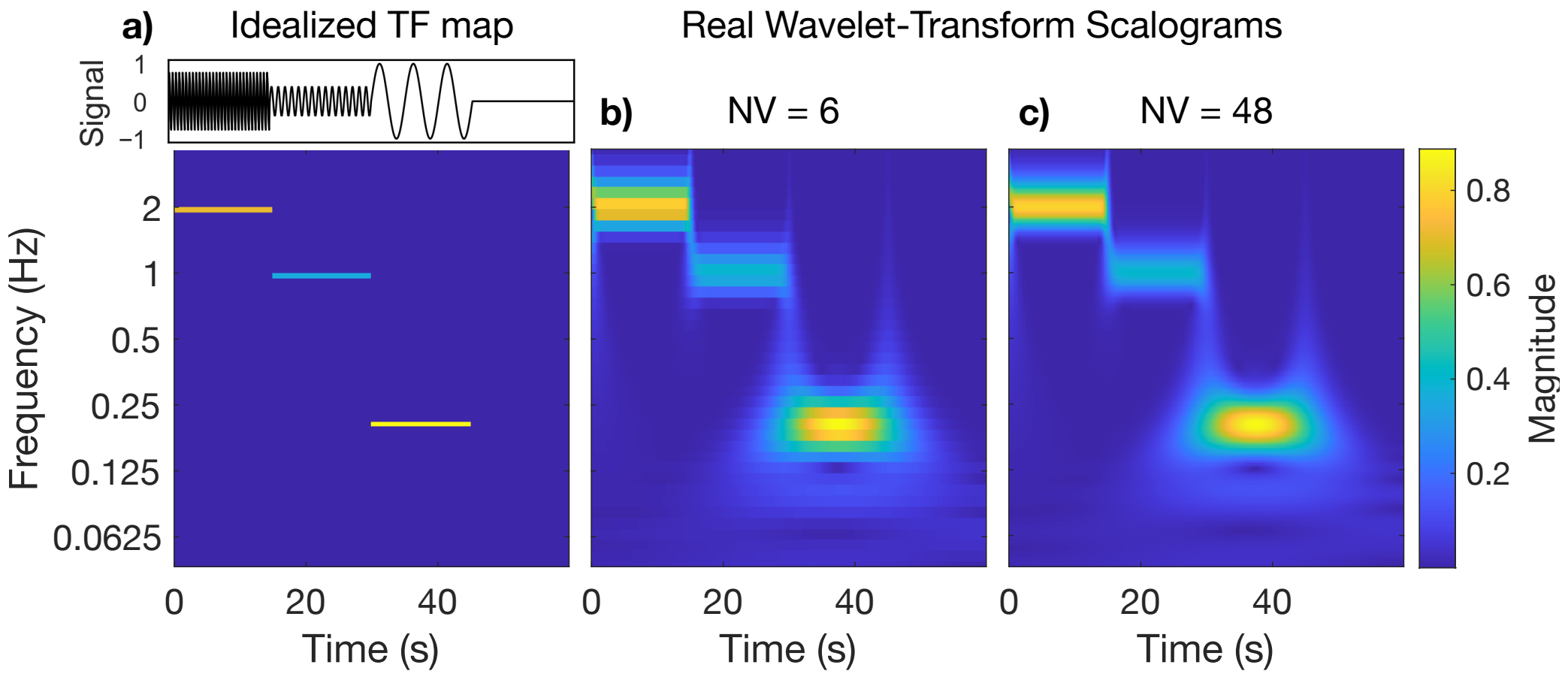

Figure S5. Time–frequency uncertainty in the continuous wavelet transform.
(a) Example signal and its idealized time–frequency structure. The signal consists of concatenated sine waves with well-defined frequencies and durations.
(b) Continuous wavelet transform scalogram computed with 6 frequency bins per one doubling of frequency (octave) (MATLAB VoicesPerOctave parameter). The oscillatory components are identifiable but appear broadened in time and frequency, reflecting intrinsic time–frequency uncertainty.
(c) Scalogram computed with 48 frequency bins per octave. Increasing the number of frequency bins produces a smoother image but does not increase the true time–frequency resolution.

S2.2 The short-time Fourier transform

The Fourier transform identifies which frequencies are present in a signal but does not indicate when those frequencies occur. A classical approach to extending Fourier analysis into the time–frequency domain is the short-time Fourier transform (STFT). The STFT estimates frequency content within short, overlapping segments of the signal, thereby providing a representation of how spectral components evolve over time.

To compute the STFT, the signal is multiplied by a window function that isolates a local portion of the data, and a Fourier transform is applied to this windowed segment. Tapered windows are used to avoid sharp boundaries, which would otherwise introduce artificial high-

frequency components in the spectrum (Cheveigné & Nelken, 2019). Sliding the window across time and repeating the transform produces a sequence of local spectra that together form a spectrogram, a time–frequency representation showing how the signal's frequency content changes over time. Figure S6 illustrates this procedure.

The time–frequency tradeoff, which is inherent to all time–frequency methods, is also evident in this example. When the Fourier transform is applied to the entire signal (panel b), the 2 Hz component is identified with high frequency precision. In contrast, when the analysis is restricted to a short window containing only a local portion of the signal, the resulting spectral peak becomes broader (panel f). This reflects increased uncertainty in the frequency estimate: a shorter observation window provides better temporal localization but reduces frequency resolution. The finite window length, therefore, determines how precisely frequencies can be estimated. As shown in panel (d), the window function itself has a characteristic width in the frequency domain. When the signal is multiplied by the window, the resulting spectral peak acquires a similar width. Once the window width is fixed, the frequency resolution of the STFT is therefore determined.

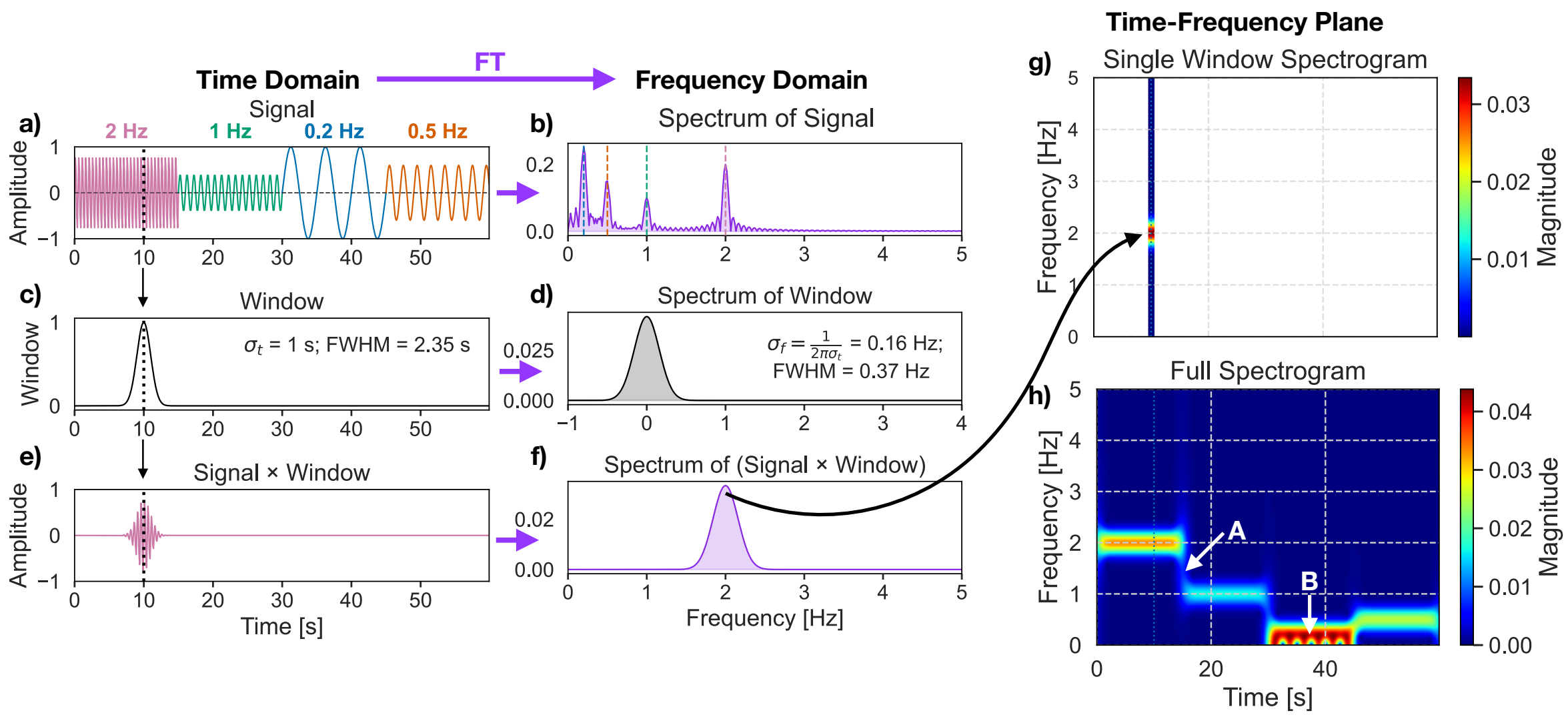

Figure S6. Illustration of the classical Fourier transform (FT) and the short-time Fourier transform (STFT).

(a) Example signal whose frequency and amplitude change over time.

(b) FT of the entire signal, showing the frequencies present but not when they occur.

(c) Gaussian window centered at t =10.

(d) Spectrum of the window function, whose width determines the frequency resolution of the STFT.

(e) Windowed segment of the signal obtained by multiplying the signal with the Gaussian window.

(f) FT of the windowed segment, revealing the local oscillation near 10 s but with a

broadened spectral peak, since the frequency resolution is constrained by the (temporal) window size.

(g) Local spectrum represented as a single column in the spectrogram, with magnitude encoded by color.

(h) Full spectrogram obtained by sliding the window across time. (A) Frequency transitions appear smeared because the window overlaps adjacent oscillations, and (B) slow oscillations may produce rippling artifacts when the window is too short to capture multiple cycles.

S2.3 Fixed vs adaptive time–frequency resolution

All time–frequency decomposition methods operate under the time–frequency tradeoff described above: improving temporal precision necessarily reduces frequency precision, and vice versa. What distinguishes different methods is how they implement this compromise between time and frequency resolution.

The STFT provides the classical solution. It analyzes the signal using a window of fixed duration that is slid across time. For each windowed segment, a Fourier transform is computed, which evaluates the entire spectrum of frequencies within that window. Because all frequencies are estimated from the same time segment, they share the same temporal and frequency resolution. This becomes problematic when analyzing signals that contain both slow and fast oscillations. If the window is chosen long enough to capture several cycles of low-frequency oscillations, high-frequency components are analyzed with windows that are unnecessarily long relative to their period. Conversely, if a short window is chosen to maximize temporal precision and capture rapid oscillations, the window may contain only a small fraction of a low-frequency cycle, making it difficult to estimate its frequency accurately.

Wavelet transforms address this limitation by adapting the analysis window to the frequency of interest. In the wavelet framework, the “window” corresponds to the temporal width of the wavelet itself. The commonly used Morlet wavelet consists of a sinusoid modulated by a Gaussian envelope (with minor corrections for admissibility that are negligible in practice) and can therefore be viewed as analogous to an STFT with a Gaussian window. The wavelet transform analyzes a signal using scaled versions of a base waveform (the mother wavelet): stretching the wavelet yields lower-frequency components with longer temporal support, whereas compressing it produces higher-frequency components with shorter support. In this way, wavelet scaling naturally provides window sizes that adapt to the time–frequency resolution requirements.

This scaling ensures that the analysis window captures approximately the same number of oscillatory cycles at every frequency. Consequently, the relative frequency resolution remains constant across scales. In signal processing terms, this property is often described as a constant relative bandwidth, meaning that the ratio between a wavelet's center frequency and its bandwidth remains approximately constant.

The resulting tiling of the time–frequency plane, therefore, differs fundamentally from that of the STFT (Figure S7). Instead of using equal-duration windows at all frequencies, wavelet analysis uses long windows for low frequencies and short windows for high frequencies, allowing the transform to balance temporal and spectral resolution across a wide range of time scales.

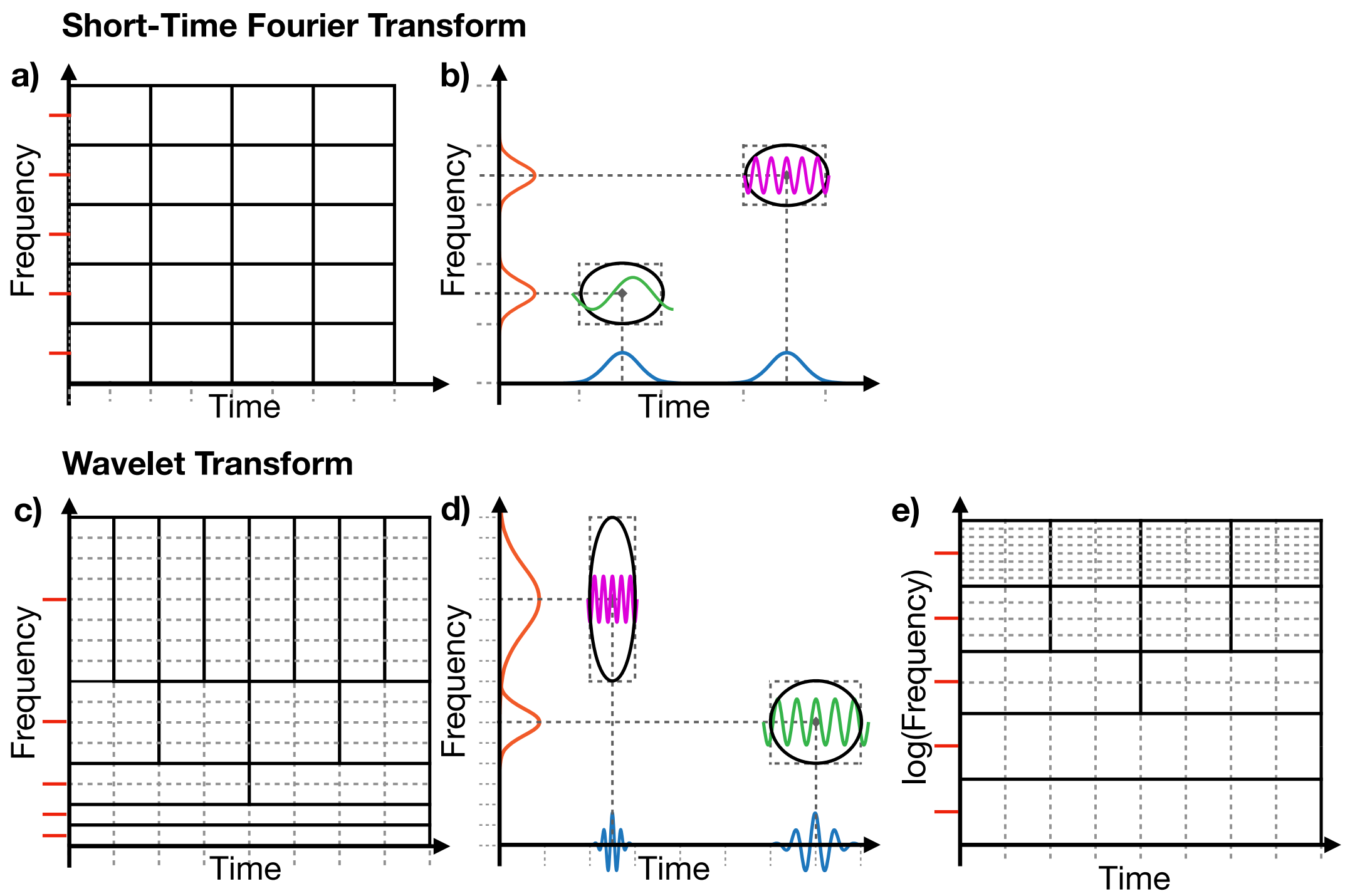

Figure S7. Comparison of time–frequency resolution in the short-time Fourier transform (STFT) and the wavelet transform (WT).

(a) Time–frequency tiling of the STFT. Because the analysis window has a fixed duration across all frequencies, the time–frequency plane is partitioned into equal-sized rectangles. Each rectangle represents the temporal and spectral region over which the signal is analyzed.

(b) Example STFT windows at low and high frequencies. The fixed window width imposes the same temporal extent at all frequencies. Consequently, high-frequency oscillations contain many cycles within the window, whereas low-frequency oscillations may contain only a fraction of a cycle, reducing frequency resolution. Gaussian time windows are shown along

the x-axis (blue), with their corresponding Gaussian spectra along the y-axis (orange), for the illustrated time–frequency points.
(c) Time–frequency tiling of the WT. Each rectangle represents the temporal and spectral support of a scaled wavelet. Low-frequency wavelets are longer in duration and narrower in frequency bandwidth, whereas high-frequency wavelets are shorter in duration and broader in bandwidth.
(d) Example WT windows at low (green) and high (pink) frequencies. Scaling the wavelet changes its temporal and spectral extent while preserving approximately the same number of oscillatory cycles within the window. Morlet wavelets at the illustrated time–frequency points are shown along the x-axis (blue), with their corresponding Gaussian spectral envelopes along the y-axis (orange).
(e) WT tiling from panel (c) displayed on a logarithmic frequency axis. Because wavelet center frequencies are geometrically spaced, the tiles appear evenly distributed along the frequency axis, reflecting the approximately constant relative bandwidth of the transform.

S3 The Morlet Wavelet and Its Properties

Wavelet-based coherence analyses in recent fNIRS hyperscanning studies are most commonly implemented in MATLAB. Many studies relied on the Cross Wavelet and Wavelet Coherence Toolbox (Grinsted et al., 2004), which implements the methodology of Torrence & Compo (1998). Equivalent functionality is now available in MATLAB's native Wavelet Toolbox (MathWorks, 2024), which provides a well-supported and convenient alternative.

In this section, we describe how the Wavelet Toolbox (MATLAB R2024b) implements the CWT using the analytic Morlet wavelet (`'Wavelet' = 'amor'`), which is the default wavelet used in the function that calculates the WTC (`wcoherence`). We present the mathematical formulation used internally, explain how scale is mapped to physical frequency, and show how the time–frequency tradeoff is determined by the wavelet parameters. We further derive practical quantities useful for scalogram interpretation, including the effective frequency resolution, the e-folding time, and the cone of influence. Where relevant, we relate MATLAB's implementation to the formulation of Torrence & Compo (1998) and the Grinsted et al. (2004) toolbox.

S3.1 Frequency-domain formulation

S3.1.1 Why start with a frequency-domain formulation?

In MATLAB's Wavelet Toolbox, the Morlet wavelet is implemented directly in the frequency domain, reflecting how the wavelet transform (WT) is usually computed in practice.

Conceptually, the WT can be understood as a convolution in the time domain, in which the wavelet is slid across the signal and compared with it at each time point. Computing this convolution directly, however, is computationally expensive.

Instead, one can exploit the Convolution Theorem, which states that convolution in time is equivalent to pointwise multiplication in the frequency domain. Therefore, instead of carrying out the convolution directly, the signal is first transformed into the frequency domain using the Fast Fourier Transform (FFT), and the wavelet is constructed in the frequency domain as well. The two spectra are then multiplied, and the result is transformed back into the time domain using the inverse FFT. This yields the WT coefficients.

Because these Fourier-based operations are highly efficient, this approach is typically much faster than computing the convolution directly (see also Torrence and Compo, 1998; Grinsted et al., 2004).

S3.1.2 Wavelet spectrum in dimensionless frequency

In MATLAB's Wavelet Toolbox one of the first steps of the CWT is the creation of a filterbank, via the function `cwtfilterbank`. This is a collection of all scaled versions of the mother wavelet that will be used to analyze the signal.

Inspection of the source code (specifically the function `wavelet.internal.cwt.wavbpfilters` used by `cwtfilterbank` with the parameter `'Wavelet' = 'amor'`), shows that the mother wavelet is defined as a function of a frequency variable Ω. At first glance, this notation can be confusing because Ω does not correspond to ordinary frequency in Hertz. Instead, it represents digital angular frequency, measured in radians per sample. This quantity describes how much the phase of an oscillation advances between consecutive samples of the discretely sampled signal. Defining the wavelet in these dimensionless units makes the implementation independent of the sampling rate. Once the sampling frequency of the signal is specified, these frequencies can be converted to physical units such as Hertz.

With this notation, the analytic Morlet wavelet at scale s is defined in the frequency domain as a Gaussian envelope restricted to positive frequencies:

$$\widehat{\psi_s}(\Omega) = 2\ \exp\left(-\frac{(s\Omega-\omega_0)^2}{2}\right) U(\Omega),$$

which can equivalently be written in standard Gaussian form as

$$2\ \exp\left(-\frac{\left(\Omega-\frac{\omega_0}{s}\right)^2}{2\left(\frac{1}{s}\right)^2}\right) U(\Omega).$$

Here:

- ω_0 is the angular center frequency of the mother wavelet (at scale $s = 1$) (also called the *Morlet wavelet parameter*). In `wavelet.internal.cwt.wavbpfilters` this parameter is set to be 6: `fc = 6`.
- $U(\Omega)$ is the Heaviside unit step function, which is zero for $\Omega \leq 0$ and one otherwise.
- *s* is the scale parameter, which stretches or compresses the mother wavelet in time. In this formulation, scale is unitless, whereas Torrence and Compo (1998) treat scale as having units of time; the conversion between the two definitions is determined by the sampling interval ($\Delta t \ = \ f_s$). This leads to small differences in the formulas listed here compared to those in Torrence and Compo (1998).

The step function ensures that the wavelet contains only positive frequencies, making it an *analytic* wavelet.

Scaling the mother wavelet by a factor *s* shifts the spectral peak to

$$\omega_0/s$$

and sets the Gaussian spectral width to

$$\sigma_\Omega = 1/\mathrm{s}.$$

Increasing the scale, therefore, moves the wavelet toward lower frequencies while narrowing its bandwidth.

S3.1.3 Wavelet spectrum in physical frequency (Hz)

In the general formulation, the wavelet is defined in terms of a dimensionless frequency variable, independent of any particular signal. However, when the wavelet is applied to a discretely sampled signal, it must be related to the sampling grid of that signal. In this context, the dimensionless angular frequency Ω (measured in radians per sample) can be converted to ordinary frequency f using the sampling frequency f_s of the analyzed signal:

$$f = \frac{\Omega}{2\pi} f_s.$$

Substituting this relationship into the definition above yields

$$\widehat{\Psi_s}(f) = 2\ \exp\left(-\frac{\left(f - \frac{\omega_0 f_s}{2\pi s}\right)^2}{2\left(\frac{f_s}{2\pi s}\right)^2}\right) U(f).$$

Further we define the *peak frequency* in Hz as:

$$f_c = \frac{\omega_0 f_s}{2\pi s}\ .$$

The wavelet can be written in the standard Gaussian form

$$\widehat{\Psi_{f_c}}(f) = 2\ \exp\left(-\frac{(f-f_c)^2}{2\left(\frac{f_c}{\omega_0}\right)^2}\right) U(f).$$

Thus the *standard deviation* (spread) in Hz is:

$$\sigma_f = \frac{f_c}{\omega_0} = \frac{f_s}{2\pi s}.$$

S3.2 Mapping wavelet scale to frequency

The continuous wavelet transform (CWT) is formally a time–scale representation. In practice, the transform is computed over a grid of scales: the mother wavelet is stretched or compressed to each scale, forming a bank of analyzing wavelets that are compared with the signal. Larger scales correspond to longer wavelets and therefore slower oscillations, while smaller scales correspond to shorter wavelets and faster oscillations.

For interpretation, however, it is usually more convenient to express the results in frequency units. This requires associating each scale with a representative frequency. Such a mapping is possible when the wavelet has a well-localized spectrum. The Morlet wavelet satisfies this condition because its Gaussian spectrum is concentrated around a single peak, allowing each scale to be associated with a characteristic frequency.

In the previous section, we derived the peak frequency of the Morlet wavelet,

$$f_c = \frac{\omega_0 f_s}{2\pi s}\ .$$

by converting the center of the wavelet spectrum from dimensionless units to Hertz. This expression defines the scale–frequency mapping used in MATLAB's Wavelet Toolbox, where the frequency associated with a scale is taken to be the peak of the wavelet spectrum.

S3.2.1 Fourier Factor (MATLAB definition)

MATLAB expresses this relationship using the Fourier factor

$$\mathrm{FF} = \frac{\omega_0}{2\pi},$$

which converts scale s to peak frequency:

$$f_c = \frac{\mathrm{FF}}{s} f_s.$$

S3.2.2 Fourier Wavelength (Torrence & Compo Definition)

Torrence & Compo (1998) define the wavelet frequency slightly differently. Rather than identifying the frequency with the peak of the wavelet's spectrum, they define it in terms of how the wavelet responds to a pure sinusoidal signal. Specifically, they ask: *if a wavelet with scale s_k is used to analyze sinusoidal signals of different frequencies, at which frequency is the resulting wavelet transform coefficient largest?*

Their analytical derivation for the Morlet wavelet yields the Fourier wavelength:

$$\lambda = \frac{4\pi}{\omega_0 + \sqrt{2+\omega_0^2}} s.$$

Note that Torrence & Compo define scale in units of time, whereas MATLAB uses a dimensionless scale. In MATLAB notation, the representative frequency of a wavelet with scale s per the Torrence & Compo Definition is:

$$f_{TC} = f_s/\lambda.$$

Sketch of the derivation

To outline the derivation, consider a sinusoidal signal (plane wave) with frequency Ω_k

$$x(t) = e^{i\Omega_k t},$$

whose Fourier transform is

$$\hat{x}(\Omega) = \delta(\Omega - \Omega_k).$$

where δ(·) denotes the Dirac delta function.

The continuous wavelet transform can be evaluated in the frequency domain, where the signal spectrum $\hat{x}$ is multiplied by the scaled wavelet spectrum $\widehat{\psi_s}^*$.

$$W_x(s,\tau) = \frac{1}{2\pi}\int \hat{x}(\Omega)\widehat{\psi_s}^*(\Omega)e^{i\Omega\tau}\mathrm{d}\Omega$$

Because the signal spectrum is a delta function, the integral simply evaluates the wavelet spectrum at the sinusoid frequency Ω_k. The resulting wavelet coefficient is therefore proportional to

$$W_x(s) \propto \widehat{\psi_s}(\Omega_k).$$

For the Morlet wavelet,

$$\widehat{\psi_s}(\Omega_k) \propto \exp\left(-\frac{(s\Omega_k-\omega_0)^2}{2}\right).$$

The strength of the wavelet response is measured as wavelet power, defined as the squared magnitude of the coefficient,

$$|W_x(s)|^2.$$

Accounting for the scale normalization of the CWT, the power is proportional to

$$|W_x(s)|^2 \propto \mathrm{s}\exp(-(s\Omega_k - \omega_0)^2)\ .$$

The scale that produces the strongest response is obtained by differentiating this expression with respect to *s* and setting the derivative to zero. Solving the resulting equation yields

$$s_k = \frac{1}{2}\frac{\omega_0 + \sqrt{2+\omega_0^2}}{\Omega_k}.$$

Converting angular frequency to Hertz using

$$f_k = \Omega_k f_s/(2\pi)$$

yields

$$f_k = \frac{\omega_0 + \sqrt{2+\omega_0^2}}{4\pi s_k} f_s = \frac{f_s}{\lambda_k}.$$

Thus, the frequency associated with a wavelet scale is defined as the frequency of the sinusoid that produces the maximum wavelet response at that scale.

S3.2.3 Comparison of the two definitions for $\boldsymbol{\omega_0 = 6}$

For the commonly used parameter $\omega_0 = 6$, the two definitions yield very similar scale–frequency relationships:

- MATLAB peak-frequency definition:

$$f_c \approx (1/1.045\ s)\, f_s,$$

- Torrence & Compo's Fourier-wavelength definition:

$$f_{TC} \approx (1/1.033\ s)\, f_s.$$

In this case, both definitions provide nearly identical mappings between scale and physical frequency. In addition, a wavelet's characteristic frequency is approximately the inverse of its scale:

$$f \approx \frac{1}{s}.$$

S3.3 Time-domain formulation

Although the continuous wavelet transform is commonly implemented in the frequency domain, it is still useful to examine the wavelet in the time domain to understand its temporal resolution. In MATLAB, time-domain wavelets can be obtained from a `cwtfilterbank` object using the function `wavelets(fb)`. Internally, these wavelets are generated numerically by applying an inverse Fourier transform to the analytically defined frequency-domain wavelets $\widehat{\psi_s}(f)$.

Because the Morlet wavelet has a Gaussian form in the frequency domain, its time-domain expression can be derived analytically. Consider a Gaussian spectrum centered at f_c with standard deviation σ_f:

$$\widehat{\psi_{f_c}}(f) = 2\exp\left(-\frac{(f-f_c)^2}{2\sigma_f^2}\right).$$

Using the inverse Fourier transform convention:

$$\psi(t) = \int_{-\infty}^{\infty} \hat{\psi}(f)\, e^{i2\pi ft}\, df,$$

the inverse transform of this Gaussian yields

$$\psi_{f_c}(t) = \sigma_f\, 2\sqrt{2\pi}\exp\left(-\frac{t^2}{2\sigma_t^2}\right) e^{i2\pi f_c t},$$

where

$$\sigma_t = \frac{1}{2\pi\sigma_f}$$

is the temporal standard deviation.

Thus, the time-domain Morlet wavelet can be interpreted as a *complex sinusoid* at frequency f_c:

$$e^{i2\pi f_c t} = \cos(2\pi f_c t) + i\sin(2\pi f_c t),$$

that is localized in time by a *Gaussian envelope*:

$$\exp\left(-\frac{t^2}{2\sigma_t^2}\right).$$

Strictly speaking, the analytic Morlet wavelet is not a perfect Gaussian in the frequency domain because negative frequencies are removed by the step function $U(f)$. This modification introduces a small correction to the exact time-domain form we derived above. When the center frequency is sufficiently larger than the bandwidth, this correction becomes negligible. In practice, with the commonly used parameter $\omega_0 = 6$, the negative-frequency

tail lies several standard deviations away from the center frequency, making the Gaussian approximation effectively exact.

In addition, wavelets must satisfy the admissibility condition (Farge, 1992), which implies that the wavelet must have zero mean. This property ensures that the wavelet transform responds to oscillatory structure and temporal changes in the signal rather than to its average level. The Morlet wavelet derived above does not strictly meet this requirement and can be corrected by subtracting a constant term:

$$\psi_{f_c}(t) \propto \exp\left(-\frac{t^2}{2\sigma_t^2}\right)\left[e^{i2\pi f_c t} - e^{-\omega_0{}^2/2}\right]$$

For typical choices $\omega_0 \geq 5$, the correction term $e^{-\omega_0{}^2/2}$ is negligible. Thus, defining the Morlet wavelet as an analytic Gaussian in the frequency domain with $\omega_0 = 6$ yields a wavelet that is effectively admissible and whose time-domain form is well approximated by a Gaussian-modulated complex sinusoid.

Figure S8 illustrates Morlet wavelets centered at several frequencies in both the time and frequency domains.

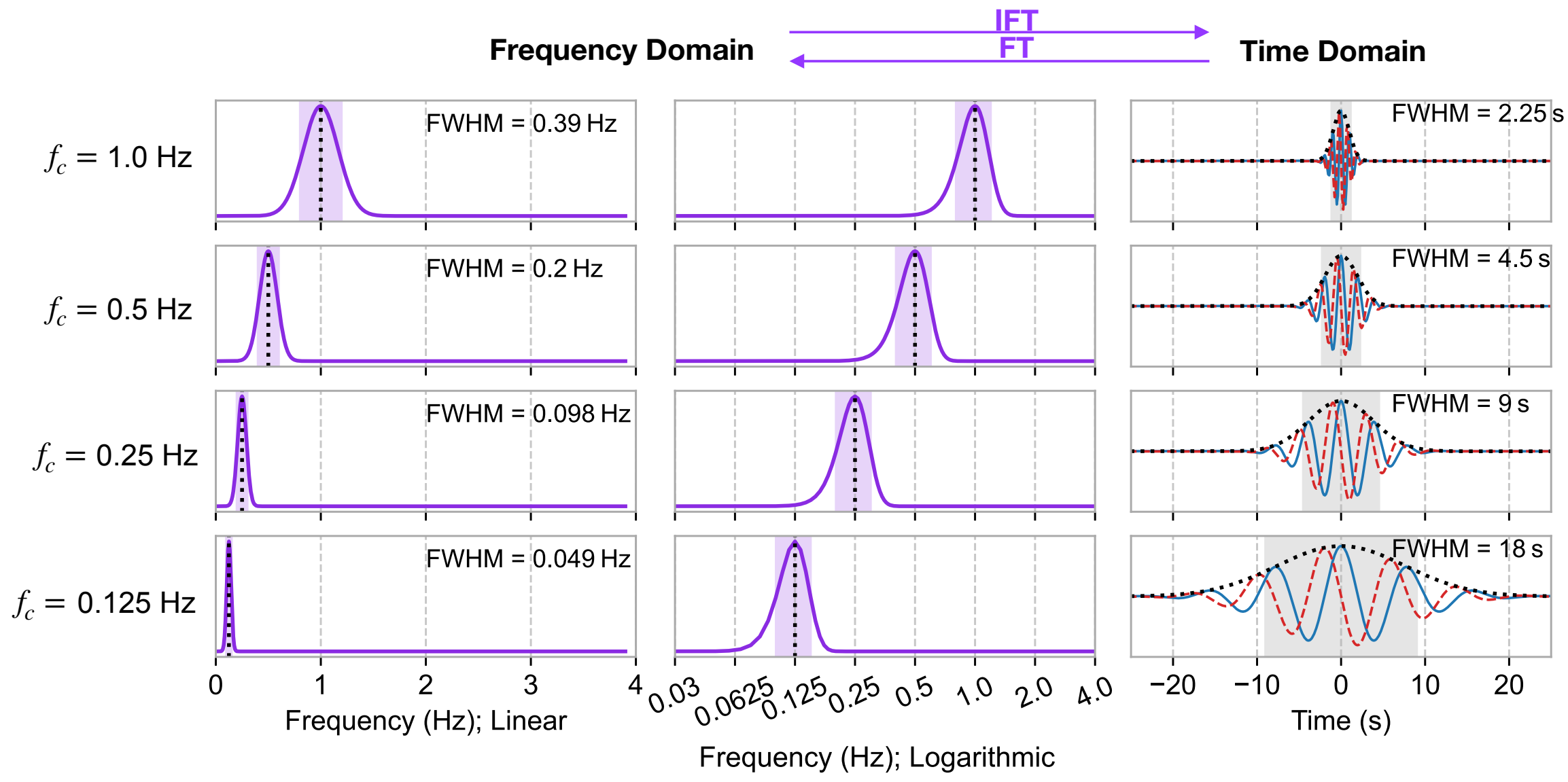

Figure S8. Example complex Morlet wavelets in a filterbank.

Wavelets spaced one octave (doubling of frequency) apart are shown in the frequency domain on linear (left) and logarithmic (middle) axes, and in the time domain (right). Shaded regions indicate the full width at half maximum (FWHM) in frequency (purple) and time (gray). On a linear frequency axis, low-frequency wavelets appear narrow and high-frequency wavelets broad because the absolute bandwidth increases with center frequency. When plotted on a logarithmic frequency axis, however, all wavelets appear equally wide because they have constant relative bandwidth (constant-Q): each wavelet spans the same

proportion of frequencies around its center frequency. Only four wavelets are shown for illustration; in a full CWT, many densely spaced wavelets are used, whose substantial overlap in frequency produces smooth but highly redundant scalograms.

S3.4 Normalization conventions

Normalization defines how the amplitude of the wavelet changes with scale. In the present derivation, the normalization constant of the time-domain wavelet is

$$\sigma_f 2\sqrt{2\pi},$$

which is proportional to the inverse of scale, since $\sigma_f \propto \frac{1}{s}$.

This normalization follows directly from how the wavelet was defined in the frequency domain: wavelets at all scales were constructed to have the same peak amplitude (equal to 2). As a result, the corresponding time-domain wavelets inherit a $1/s$ scaling factor. This is commonly referred to as L1 normalization or amplitude normalization.

With this choice, oscillations of *equal amplitude* in the signal produce comparable magnitudes in the scalogram across frequencies. Consider a signal composed of two sinusoidal components with equal amplitudes but different frequencies: under amplitude normalization, the magnitude of the wavelet coefficients at both component frequencies will be equal.

An alternative convention is used by Torrence & Compo (1998), and in the implementation of Grinsted et al. (2004), where the wavelets are L2 normalized, i.e., scaled by $1/\sqrt{s}$. This normalization equalizes energy (or power) across scales rather than amplitude, leading to different visual characteristics in the scalogram. In the previous example, the higher frequency component will produce a stronger WT magnitude compared to the lower frequency. For broader discussions on wavelet normalization, see Moca et al. (2021).

Importantly, while these normalization differences change the appearance of WT scalograms, they do not affect the WTC, as scale-dependent normalization factors cancel out when the cross-spectrum is normalized with individual spectra.

S3.5 Time-frequency resolution

The widths of the wavelet in time and frequency determine the precision with which the transform can localize oscillatory activity. For the Morlet wavelet, these widths correspond to the standard deviations of the Gaussian envelope in time (σ_t) and frequency (σ_f).

The frequency spread σ_f describes how narrowly the wavelet is tuned in frequency and therefore reflects its frequency resolution. The temporal spread σ_t describes the duration of the wavelet in time and therefore determines its temporal resolution.

S3.5.1 Time-frequency tradeoff

For the Morlet wavelet, the temporal and spectral spreads are related by

$$\sigma_t = \frac{1}{2\pi\sigma_f}.$$

This relationship expresses the time–frequency tradeoff: improving frequency resolution (smaller σ_f) necessarily increases temporal spread (larger σ_t), and vice versa.

Multiplying both sides by σ_f gives

$$\sigma_t\sigma_f = \frac{1}{2\pi}.$$

The Heisenberg–Gabor uncertainty principle states that the product of time and frequency uncertainties cannot fall below a theoretical lower bound (Gabor, 1946). When uncertainties are defined using standard deviations, this limit is

$$\sigma_t\sigma_f \geq \frac{1}{4\pi}.$$

Because admissible wavelets must have (approximately) zero mean, no wavelet can achieve this theoretical minimum exactly (Farge, 1992). The Morlet wavelet, however, consists of a sinusoid modulated by a Gaussian envelope (with a negligible correction for mathematical admissibility), and Gaussian functions uniquely attain the minimum uncertainty product. As a result, the Morlet wavelet approaches this lower bound and is therefore nearly optimally concentrated in both time and frequency.

S3.6 Practical measures of wavelet width

Although Gaussian functions extend infinitely in time and frequency, most of their energy is concentrated near the center. For practical interpretation, it is therefore useful to define effective widths.

S3.6.1 Full Width at Half Maximum (FWHM)

A common measure is the full width at half maximum (FWHM), defined as the distance between the points where the Gaussian falls to half of its peak value. For a Gaussian,

$$\text{FWHM} = 2\sqrt{2\ln 2}\ \sigma \approx 2.355\ \sigma.$$

The effective bandwidth of the Morlet wavelet (width in frequency) can be written in terms of peak frequency f_c,

$$\mathrm{FWHM}_f \approx 2.355\,\sigma_f = 2.355\,\frac{f_c}{\omega_0}.$$

This quantity provides a practical estimate of the frequency range over which the wavelet responds strongly.

Similarly, the effective temporal width can be defined as

$$\mathrm{FWHM}_t = 2\sqrt{2\ln 2}\,\sigma_t \approx 2.355\,\sigma_t.$$

S3.6.2 E-folding time

Another commonly used measure of a wavelet's temporal width is the *e-folding time* (Torrence & Compo, 1998). It is defined as the time at which the wavelet power falls to $1/e^2$ (≈13.5%) of its maximum value.

We can derive the e-folding time of a Gaussian analytically.

Consider the standard Gaussian

$$g(t) = e^{-\frac{t^2}{2\sigma^2}}.$$

The e-folding time is the time at which the power $|g(t)|^2$ decays to $1/e^2$ of its maximum (which occurs at $t = 0$):

$|g(\tau_e)|^2 = e^{-2}|g(0)|^2.$

Substituting the expression for $g(t)$ gives

$$e^{-\frac{\tau_e^2}{\sigma^2}} = e^{-2}.$$

Equating exponents yields

$$\frac{\tau_e^2}{\sigma^2} = 2,$$

and therefore

$$\tau_e = \pm\sqrt{2}\,\sigma.$$

Thus, for a Gaussian centered at zero, the points $\tau_e = \pm\sqrt{2}\sigma$ mark the locations where its power has decayed to 13.5% of the peak.

Since the Morlet wavelet's envelope in time is Gaussian with standard deviation σ_t, its e-folding time is

$$\tau_e = \sqrt{2}\,\sigma_t.$$

Expressing σ_t in terms of scale s yields:

$$\tau_e = \sqrt{2}\,s/f_s,$$

where f_s is the sampling frequency.

This is exactly related to the derivation of Torrence and Compo (1998), who state that the e-folding time for a Morlet wavelet is $\sqrt{2}\,s$. The additional factor f_s again reflects the fact that we defined scale as a unitless quantity, whereas Torrence and Compo define it in units of time, i.e., relative to the sampling frequency.

Figure S9 illustrates these measures of temporal width. The half-maximum points lie at: $\pm\sqrt{2\ln 2}\,\sigma_t \approx \pm 1.18\,\sigma_t$. Red vertical lines mark the e-folding time, at $\pm\sqrt{2}\,\sigma_t \approx \pm 1.41\,\sigma_t$.

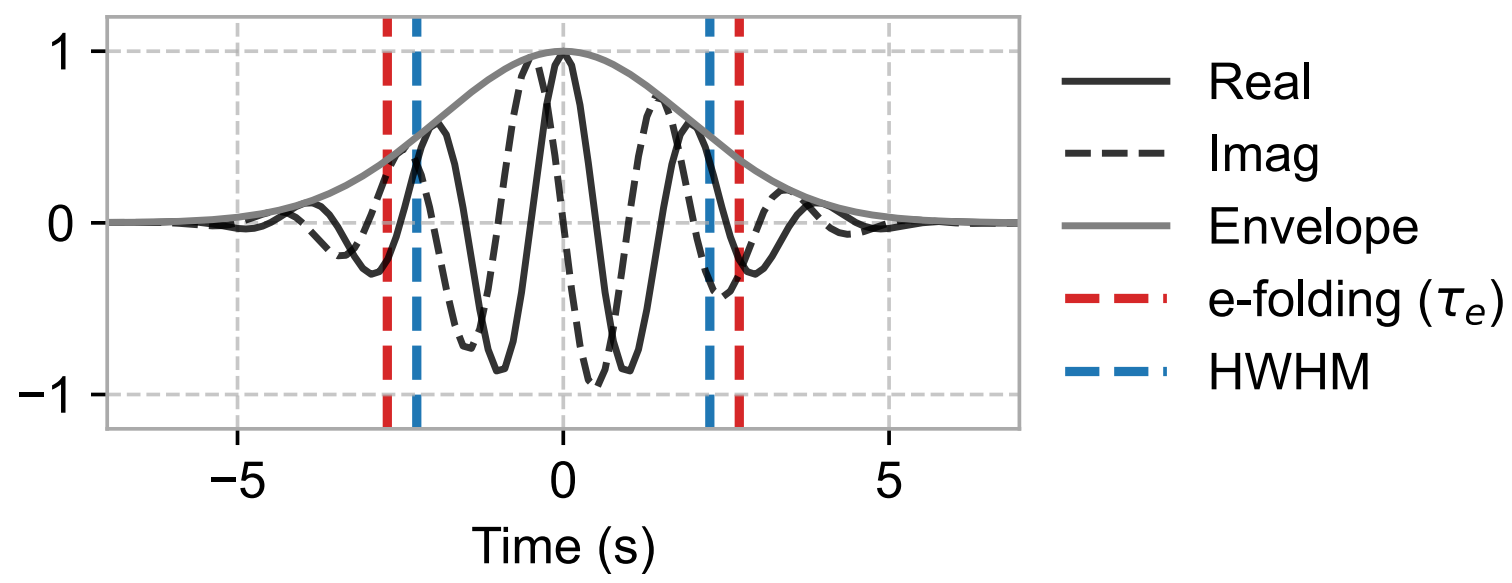

Figure S9. Temporal extent of the Morlet wavelet.
Morlet wavelet in the time domain. Blue vertical lines indicate the half-width at half-maximum (HWHM) of the Gaussian envelope; the full width at half maximum (FWHM) spans between these lines. Red vertical lines mark the e-folding time, the points at which the wavelet power has decayed to e^{-2} (≈13.5%) of its peak value.

S3.6.3 Estimating temporal uncertainty for $\omega_0 = 6$

For a Morlet wavelet with $\omega_0 = 6$ centred at a frequency f_c, the standard deviation in time is

$$\sigma_t = \frac{\omega_0}{2\pi f_c} = \frac{6}{2\pi f_c} \approx \frac{0.955}{f_c}.$$

Since this is close to $1/f_c$, it is sometimes approximated as one characteristic period of the wavelet, $P_c = 1/f_c$.

The e-folding time is then

$$\tau_e = \sqrt{2}\sigma_t = \sqrt{2}\frac{\omega_0}{2\pi f_c} \approx \frac{1.35}{f_c} = 1.35 P_c.$$

Each WT coefficient therefore draws on a temporal neighbourhood of width $2\tau_e \approx 2.7/f_c$, centred on its time point. As an example, consider a frequency range of interest from 0.02 Hz to 0.1 Hz (periods 50-10 s). At the lowest frequency, a coefficient pools contributions across

$$2\tau_e \approx \frac{2 \cdot 1.35}{0.02\ \text{Hz}} = 135\ \text{s},$$

while at the highest frequency the same window narrows to

$$2\tau_e \approx \frac{2 \cdot 1.35}{0.1\ \text{Hz}} = 27\ \text{s}.$$

S3.7 Constant-Q property

The bandwidth of the Morlet wavelet is proportional to its center frequency:

$$\sigma_f = \frac{f_c}{\omega_0}.$$

The ratio between bandwidth and center frequency therefore remains constant across scales:

$$\frac{\sigma_f}{f_c} = \frac{1}{\omega_0}.$$

This property is known as constant relative bandwidth (or constant-Q). It means that each wavelet captures the same number of oscillatory cycles regardless of its center frequency. The number of cycles it captures is set by the ω_0 parameter.

S3.8 The Morlet wavelet parameter $\boldsymbol{\omega_0}$

The parameter ω_0 determines the central frequency of the mother Morlet wavelet and controls how many oscillatory cycles are contained within the wavelet envelope. It therefore directly governs the balance between temporal and frequency resolution.

From the relationships derived above, the spectral and temporal spreads of the wavelet are

$$\sigma_f = \frac{f_c}{\omega_0},\ \ \sigma_t = \frac{\omega_0}{2\pi f_c}.$$

These expressions show that increasing ω_0 reduces the spectral spread (improving frequency resolution) while increasing the temporal spread (reducing temporal precision). Conversely, smaller values of ω_0 produce shorter wavelets with broader spectral bandwidths. In this way, ω_0 determines how the inherent time–frequency uncertainty of the wavelet is distributed between time and frequency.

A commonly used value is $\omega_0 = 6$, which provides a practical compromise: the wavelet contains enough oscillatory cycles to define a meaningful center frequency while remaining sufficiently localized in time to capture transient signal features.

Figure S10 illustrates how ω_0 shapes the time–frequency properties of the Morlet wavelet.

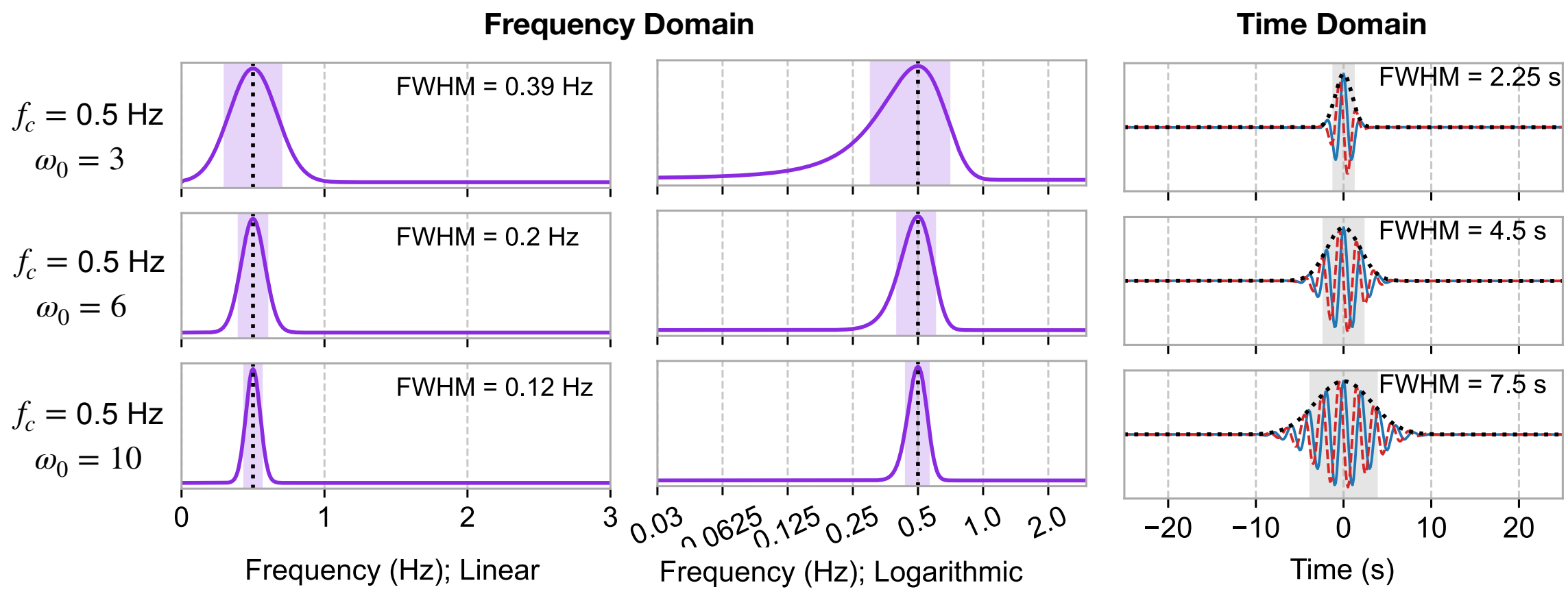

Figure S10. Effect of the ω_0 parameter on the Morlet wavelet.
Morlet wavelets constructed with three values of ω_0 (3, 6, and 10), all centered at 0.5 Hz. Smaller ω_0 values produce shorter wavelets with fewer oscillations under the Gaussian envelope and broader spectral bandwidth, whereas larger ω_0 values produce longer wavelets with more oscillatory cycles and sharper frequency selectivity.

S3.9 The Cone of Influence (COI)

The COI marks the region of the time-frequency plane in which edge effects become non-negligible. The intuition is simple: every wavelet coefficient is computed by sliding a wavelet of finite width along the signal. When the wavelet sits entirely on real data, the coefficient is trustworthy; when part of it hangs off the start or end of the signal, it overlaps padded or undefined samples, contaminating the coefficient. The COI is the boundary between these two cases: coefficients inside it are reliable, whereas coefficients outside it should be interpreted with caution.

How far this contamination reaches, that is, how many coefficients need to be discarded at the signal edges, depends on how wide the wavelet is, which in turn depends on its frequency. Following Torrence & Compo (1998), we quantify this width using the e-folding time τ_e.

For a Morlet wavelet with $\omega_0 = 6$ at frequency f_c (or period $P_c = 1/f_c$) we derived

$$\tau_e(f_c) = \sqrt{2}\sigma_t = \sqrt{2}\frac{\omega_0}{2\pi f_c} \approx \frac{1.35}{f_c} .$$

Consider a wavelet $\psi_{f_c}(t - t_0)$, which produces a WT coefficient at the time-frequency point (t_0, f_c). We deem this coefficient reliable if the wavelet's center t_0 is at least $\tau_e(f_c)$ away from both edges of the signal:

$$\tau_e(f_c) \leq t_0 \leq T - \tau_e(f_c).$$

The boundary between reliable and unreliable coefficients is obtained by taking the limiting cases in these two inequalities, in which the wavelet center sits exactly $\tau_e(f_c)$ from the edge:

$$t_{left} = \tau_e(f_c) \text{ and } t_{right} = T - \tau_e(f_c).$$

Evaluating these two expressions across all frequencies traces two symmetric curves on the scalogram, which together form the COI.

τ_e increases as f_c decreases, meaning that low-frequency wavelets are wide in time and produce many contaminated coefficients, whereas high-frequency wavelets are narrow in time and remain reliable close to the signal edges. This is illustrated in Figure S11. The higher-frequency wavelet (pink) is contained almost entirely within the signal window, so its coefficient (pink square) is reliable and lies above the COI curve. The lower-frequency wavelet (green) extends visibly beyond the signal, so its coefficient (green square) is unreliable and lies below the curve. If we evaluated the signal at even lower frequencies, τ_e would exceed half the signal length, and no coefficient would be reliable. In order to yield useful coefficients at a chosen frequency of interest f, we must ensure that the signal length is greater than $2\tau_e(f)$.

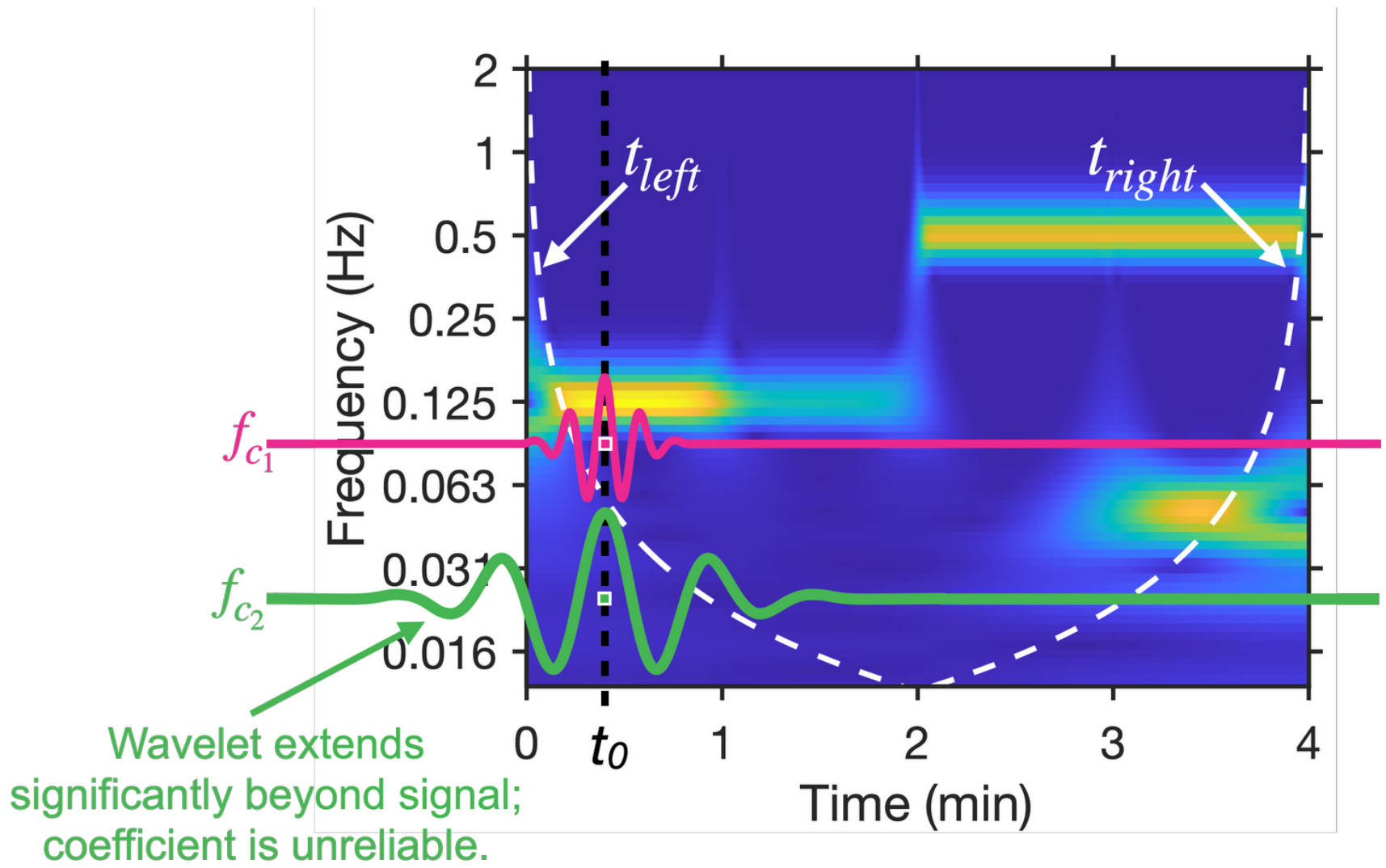

Figure S11. Cone of influence for the Morlet wavelet.

The cone of influence (white dashed curve) marks the region where boundary effects become non-negligible. Two Morlet wavelets centered at the same time point illustrate the concept. The lower-frequency wavelet (green) has a longer temporal extent and extends

beyond the signal boundary, so the corresponding coefficient lies outside the COI. The higher-frequency wavelet (pink) is shorter in time and remains largely within the signal, producing a coefficient inside the COI.

To overlay the COI on a time-frequency plot in Matlab, the e-folding time is evaluated on the frequency grid `f` and converted to left and right boundary times.

```
% --- Plot matrix ---
imagesc(ax, t, f, WT);                       % plot wavelet-transform (WT) matrix
axis(ax,'xy');                               % display high frequencies at top
set(ax,'YScale','log');                      % logarithmic frequency axis

% --- Cone of influence ---
omega0 = 6;                                  % Morlet wavelet parameter
tStart = min(t); tEnd = max(t);              % Signal edges
tau_e = (sqrt(2)*omega0) ./ (2*pi*f);        % e-folding time at each frequency
                                             % tau_e = 1.35 / f

tau_e = min(tau_e, (tEnd - tStart)/2);  % close cone in the middle of the
                                        % recording (to handle lowest freqeuncies
                                        % where no coefficients are reliable)
tLeft = tStart + tau_e;                 % left boundary (near signal start)
tRight = tEnd - tau_e;                  % right boundary (near signal end)

% --- Plot COI ---
hold(ax, 'on')
plot(tLeft, f, 'w--', 'LineWidth', 1.5) % overlay cone on the matrix
plot(tRight, f, 'w--', 'LineWidth', 1.5)
```

Sometimes it might be more convenient to ask, for a given time t, what is the lowest frequency we can still trust. Inverting the relation above gives the COI in terms of frequency as a function of time:

$$f_{\mathrm{COI}}(t) = \frac{\sqrt{2}\omega_0}{2\pi \min(t,\, T-t)} = \frac{1.35}{\min(t,\, T-t)}.$$

where $\min(t,\, \mathrm{T}-t)$ is the distance from t to the nearest boundary. At a time t, only coefficients at frequencies higher than $f_{\mathrm{COI}}(t)$ are reliable.

S4 Wavelet Transform Coherence

S4.1 Smoothing in wavelet coherence

Smoothing a time-frequency map simply means replacing each value in the map with a weighted average of its neighbors (both nearby moments in time and nearby frequencies). It

is a core computational step in the calculation of wavelet coherence: smoothing the wavelet transform spectra prior to calculating coherence ensures that the resulting coherence values are meaningful and interpretable statistics rather than trivially equal to one everywhere (Torrence and Webster, 1999). The choice of smoothing kernel shape and width is not uniquely determined by theory, and different implementations have made different choices.

Torrence and Webster (1999) propose applying smoothing separately across time (at a fixed frequency) and across frequency (at a fixed time) and argue that the natural amount of smoothing in each dimension should be matched to the characteristics of the wavelet itself. The wavelet already has its own time–frequency resolution and thus represents a kind of local average in both time and frequency; the smoothing should therefore respect the limits of resolution that the wavelet imposes. Too little smoothing produces coherence values that are artificially high and uninformative, too much blurs genuine features beyond the wavelet's inherent resolution.

S4.1.1 Smoothing in time

The Morlet wavelet has a Gaussian shape in the time domain; the natural time-smoothing kernel is therefore also Gaussian, with a width matched to the wavelet's own time-domain envelope at each scale. This is the approach specified by Torrence and Webster (1999) and implemented by both Grinsted et al. (2004) and MATLAB's `wcoherence` function (MathWorks, 2024), which applies time smoothing via multiplication by a Gaussian filter in the Fourier domain.

S4.1.2 Smoothing in frequency

Torrence and Compo (1998) proposed implementing smoothing in frequency as a moving rectangular (boxcar) average across neighboring scales, in which all contributing values are weighted equally. The critical choice is the width of this window.

One way to determine the appropriate width is through what Torrence and Compo (1998) call the *scale decorrelation length*. The underlying observation is that wavelet coefficients at two nearby frequencies (scales) tend to be highly correlated, because the corresponding wavelet functions overlap substantially in frequency space. As the separation between two scales increases, their wavelet coefficients become more independent. The scale decorrelation length, denoted δj_0, is the minimum separation in scale (measured in octaves) at which the wavelet coefficients at two scales can be considered approximately independent. Torrence and Compo (1998) approximated δj_0 empirically via Monte Carlo simulation for different wavelet shapes. For the Morlet wavelet ($\omega_0 = 6$), this yields $\delta j_0 = 0.6$.

The Grinsted et al. (2004) toolbox implements scale smoothing with a boxcar window of 0.6 octaves, consistent with Torrence and Compo (1998). MATLAB's `wcoherence` function (MathWorks, 2024) uses the same filter shape but a wider default window of one full octave.

The relevant parameters in `wcoherence` and their default values are:

- `VoicesPerOctave` (default: 12): the number of scales per octave.
- `NumScalesToSmooth` (default: equal to `VoicesPerOctave`, i.e. 12): the number of adjacent scales included in the moving average, directly controlling the width of the frequency smoothing window. Because 12 voices span exactly one octave, the default smoothing window spans one full octave.

To match the 0.6-octave smoothing width of Torrence and Compo (1998) and Grinsted et al. (2004), `NumScalesToSmooth` should be set to approximately 0.6 times `VoicesPerOctave` — that is, 7 scales when using the default of 12 voices per octave, or 6 scales when using 10 voices per octave as recommended by Grinsted et al. (2004). For example:

```
wcoherence(x, y, Fs, 'VoicesPerOctave', 10, 'NumScalesToSmooth', 6).
```

To our knowledge, no published study has systematically compared coherence estimates produced by these different smoothing conventions, and the magnitude of the resulting differences in practice remains unclear. Nevertheless, when the analysis targets a predefined frequency band of interest (as is common in fNIRS applications), a narrower smoothing width of 0.6 octaves is arguably preferable: it better confines the coherence estimate to the intended frequency range and reduces contamination from adjacent bands.

S4.2 Role of phase in smoothing and the WTC

The smoothing step in the coherence calculation averages neighboring points of the cross-wavelet transform (XWT) across both time and frequency. Because the XWT is a complex-valued quantity, this averaging incorporates both the magnitude and the phase of the cross-spectrum, where phase reflects the instantaneous timing difference between the two signals. When this phase difference is consistent across neighboring time–frequency points, contributions reinforce one another during smoothing, producing high coherence. When the phase difference varies or reverses across neighboring points, contributions cancel, reducing coherence. High coherence, therefore, requires not only shared spectral power but a stable phase relationship across the region spanned by the smoothing kernel.

Figure S12 illustrates this mechanism with a controlled example. Two pairs of synthetic signals are constructed (Panels A and B), each consisting of two sinusoidal components at 1 Hz and 0.6 Hz. The signals in the two examples differ only in their phase relationships.

In Panel A, both the 1 Hz and 0.6 Hz components of signal x are phase-shifted by 180° relative to signal y. Because this phase difference is identical across both frequencies, the smoothing kernel averages consistent contributions, and the resulting WTC shows high coherence spanning the full frequency band between the two components.

In Panel B, the phase relationships are opposite at the two frequencies: the 0.6 Hz components of x and y are in phase, while the 1 Hz components are in antiphase. When the smoothing window spans both frequency bands simultaneously, the opposing phase contributions cancel one another, producing a region of low coherence between the two component frequencies despite the signals having identical spectral power to Panel A. This demonstrates that wavelet coherence is determined by the consistency of phase differences within the smoothing kernel's footprint in the time–frequency map, not by shared spectral power alone.

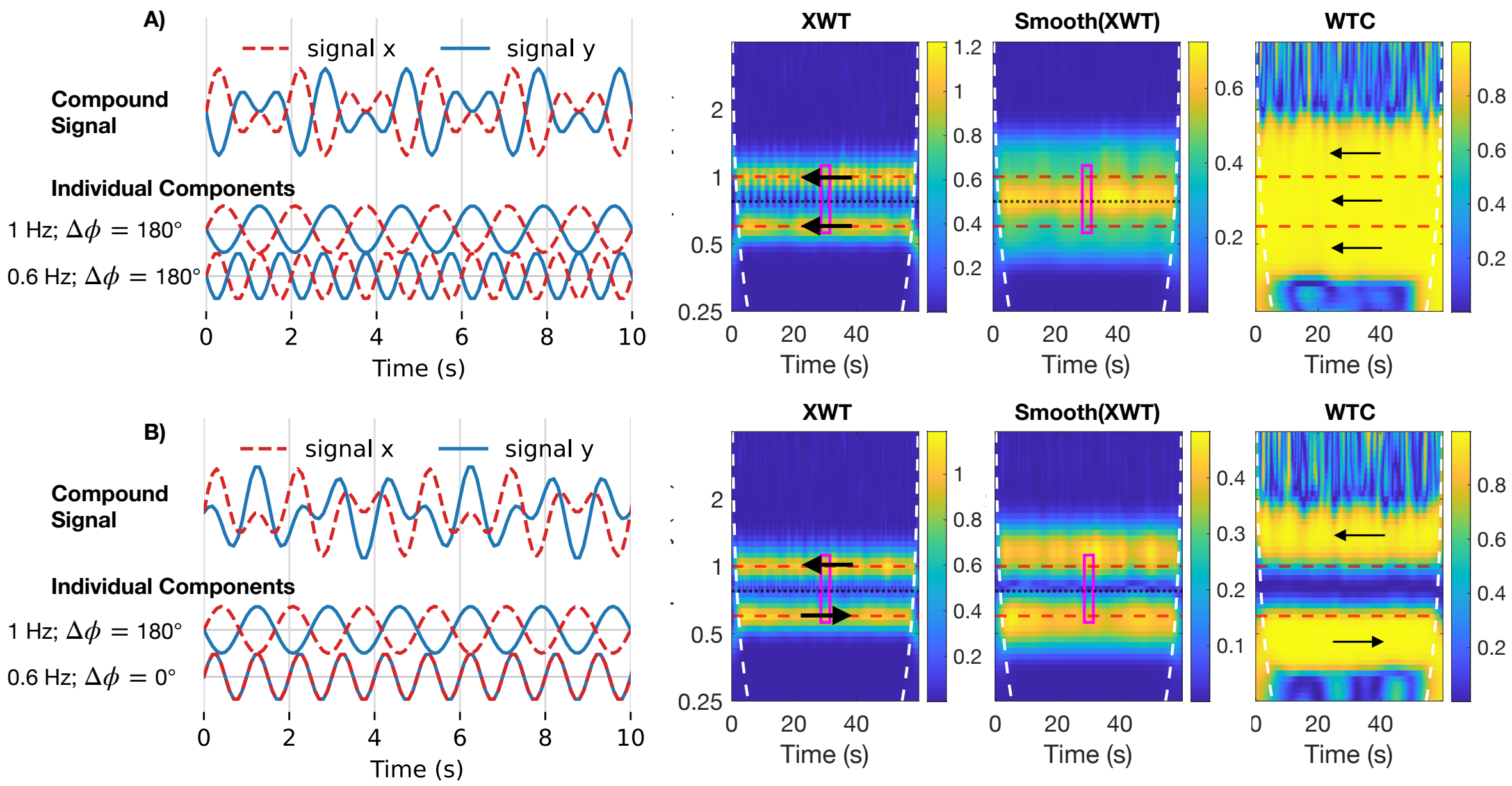

Figure S12. Smoothing and the role of phase in wavelet coherence.
Panels illustrate how wavelet coherence depends on the consistency of phase differences between two signals. Each row (A, B) shows a different pair of signals (x, y) composed of two sinusoidal components (1 Hz and 0.6 Hz). For each example, the plots display (from left to right): the signals in the time domain (with individual components shown below), the cross wavelet transform (XWT), its smoothed version, and the resulting wavelet coherence (WTC). Note that all scalograms share the y-axis of the XWT. The violet rectangle in XWT plots shows the effective smoothing area for a point at t = 30 s and f = 0.775 Hz (geometric mean of 0.6 and 1 Hz). Red dashed horizontal lines are plotted at component frequencies (1 Hz and 0.6 Hz).

A) Both components of x are phase-shifted by 180° relative to y. Because this phase difference is constant across frequencies, the smoothing step in the coherence calculation averages consistent phase relationships, resulting in a broad region of high coherence spanning the frequency band between 0.6 and 1 Hz.
B) The 0.6 Hz components of x and y are in phase, but the 1 Hz components are 180° out of phase. During smoothing, the opposite phase contributions from these two bands cancel, producing a region of low coherence between them.

S4.3 Interpreting phase relationships

In wavelet coherence plots, phase differences are commonly visualized as arrows overlaid on the time–frequency map (as in WTC plots in Figure S12), where each arrow summarizes the relative phase between the two signals across a local region of the time–frequency plane. The direction of the arrow encodes the phase difference: a rightward arrow indicates that the two signals are in phase (0°), meaning their peaks and troughs exactly coincide; a leftward arrow indicates antiphase (180°), meaning one signal is at its peak when the other is at its trough. Upward and downward arrows indicate a quarter-cycle offset (90° and 270° respectively), corresponding to one signal leading or lagging the other by one quarter of the oscillation period at that frequency. Intermediate arrow directions represent intermediate phase offsets (Figure S13).

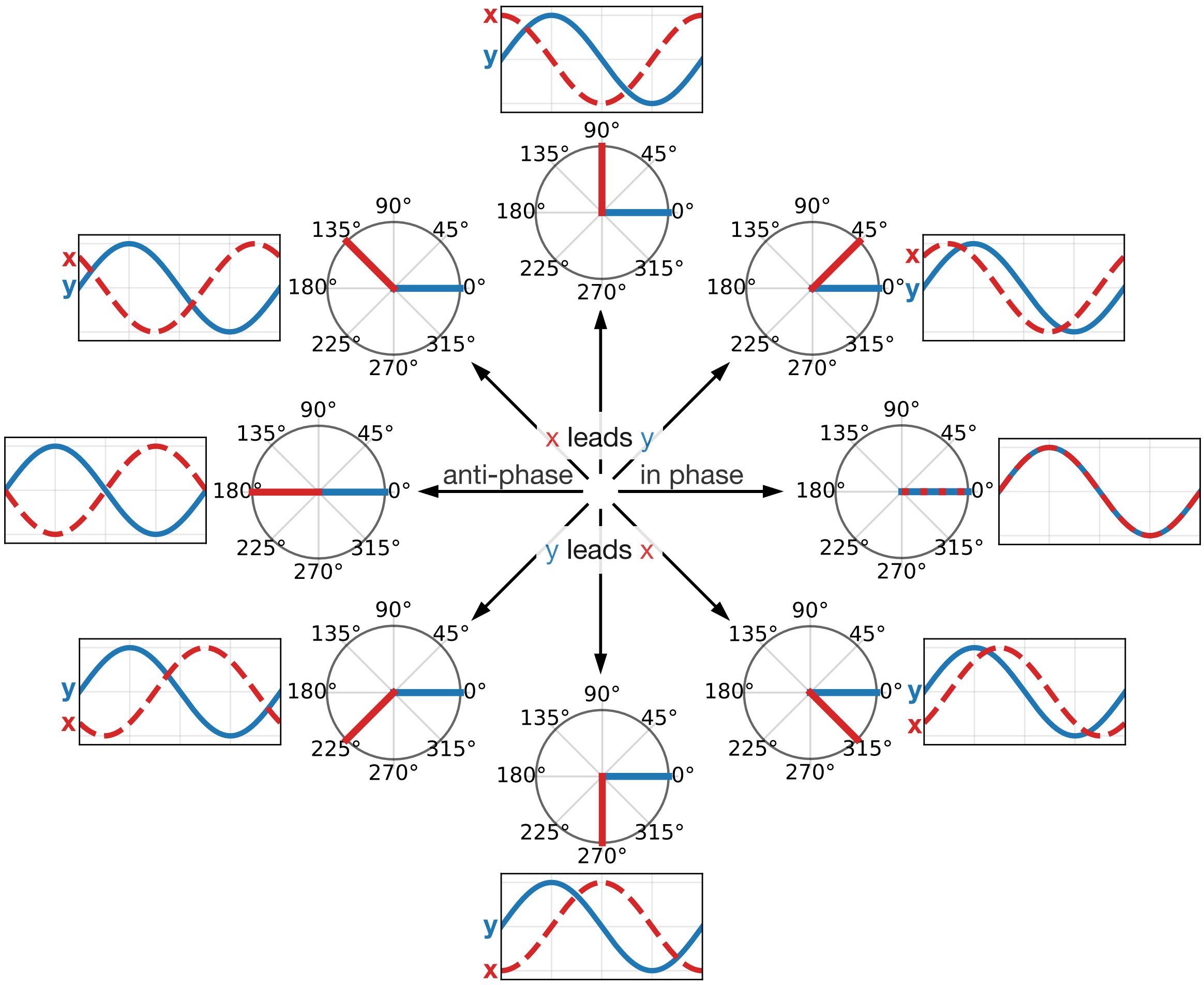

Figure S13. Interpreting phase arrows in wavelet coherence plots.

Each arrow represents a possible phase difference between two signals (x, red; y, blue) as visualized in MATLAB's wcoherence output. Arrows are shown at 0°, 45°, 90°, 135°, 180°, 225°, 270°, and 315°. The accompanying phasor diagrams depict the relative orientation of the two signals in the complex plane, where the angle between vectors indicates their phase offset. Adjacent time-domain plots show the corresponding sine waves, illustrating how these phase differences manifest in time: rightward arrows (0°) indicate in-phase coupling, leftward arrows (180°) anti-phase, upward (90°) signal x leading y by a quarter cycle, and downward (270°) x lagging by a quarter cycle.

References

Cheveigné, A. de, & Nelken, I. (2019). Filters: When, Why, and How (Not) to Use Them. *Neuron*, *102*(2), 280–293. https://doi.org/10.1016/j.neuron.2019.02.039

Farge, M. (1992). Wavelet Transforms and Their Applications to Turbulence. *Annual Review of Fluid Mechanics*, *24*(1), 395–458. https://doi.org/10.1146/annurev.fl.24.010192.002143

Grinsted, A., Moore, J. C., & Jevrejeva, S. (2004). Application of the cross wavelet transform and wavelet coherence to geophysical time series. *Nonlinear Processes in Geophysics*, *11*(5/6), 561–566. https://doi.org/10.5194/npg-11-561-2004

MacLennan, B. (2017). *Foundations of Field Computation*. https://web.eecs.utk.edu/~bmaclenn/FFC.pdf

MathWorks. (2024). *MATLAB Wavelet Toolbox (Version R2024b)*. The MathWorks, Inc., Natick, MA, USA. https://www.mathworks.com/products/wavelet.html

Moca, V. V., Bârzan, H., Nagy-Dăbâcan, A., & Mureșan, R. C. (2021). Time-frequency super-resolution with superlets. *Nature Communications*, *12*(1), 337. https://doi.org/10.1038/s41467-020-20539-9

Torrence, C., & Compo, G. P. (1998). A Practical Guide to Wavelet Analysis. *Bulletin of the American Meteorological Society*, *79*(1), 61–78. https://doi.org/10.1175/1520-0477(1998)079%253C0061:APGTWA%253E2.0.CO;2

Torrence, C., & Webster, P. J. (1999). *Interdecadal Changes in the ENSO–Monsoon System*. https://journals.ametsoc.org/view/journals/clim/12/8/1520-0442_1999_012_2679_icitem_2.0.co_2.xml
